# Large-Scale Introgression Shapes the Evolution of the Mating-Type Chromosomes of the Filamentous Ascomycete *Neurospora tetrasperma*

**DOI:** 10.1371/journal.pgen.1002820

**Published:** 2012-07-26

**Authors:** Yu Sun, Pádraic Corcoran, Audrius Menkis, Carrie A. Whittle, Siv G. E. Andersson, Hanna Johannesson

**Affiliations:** 1 Department of Evolutionary Biology, Uppsala University, Uppsala, Sweden; 2 Department of Forest Mycology and Plant Pathology, Swedish University of Agricultural Sciences, Uppsala, Sweden; 3 Department of Molecular Evolution, Uppsala University, Uppsala, Sweden; Duke University Medical Center, United States of America

## Abstract

The significance of introgression as an evolutionary force shaping natural populations is well established, especially in animal and plant systems. However, the abundance and size of introgression tracts, and to what degree interspecific gene flow is the result of adaptive processes, are largely unknown. In this study, we present medium coverage genomic data from species of the filamentous ascomycete Neurospora, and we use comparative genomics to investigate the introgression landscape at the genomic level in this model genus. We revealed one large introgression tract in each of the three investigated phylogenetic lineages of *Neurospora tetrasperma* (sizes of 5.6 Mbp, 5.2 Mbp, and 4.1 Mbp, respectively). The tract is located on the chromosome containing the locus conferring sexual identity, the mating-type (*mat*) chromosome. The region of introgression is confined to the region of suppressed recombination and is found on one of the two *mat* chromosomes (*mat a*). We used Bayesian concordance analyses to exclude incomplete lineage sorting as the cause for the observed pattern, and multilocus genealogies from additional species of Neurospora show that the introgression likely originates from two closely related, freely recombining, heterothallic species (*N. hispaniola* and *N. crassa*/*N. perkinsii*). Finally, we investigated patterns of molecular evolution of the *mat* chromosome in Neurospora, and we show that introgression is correlated with reduced level of molecular degeneration, consistent with a shorter time of recombination suppression. The chromosome specific (*mat*) and allele specific (*mat a*) introgression reported herein comprise the largest introgression tracts reported to date from natural populations. Furthermore, our data contradicts theoretical predictions that introgression should be less likely on sex-determining chromosomes. Taken together, the data presented herein advance our general understanding of introgression as a force shaping eukaryotic genomes.

## Author Summary

Introgression is a process by which genetic material from one species becomes infiltrated into another, genetically distinct species. Introgression usually occurs via sexual reproduction: individuals of two species mate and produce a hybrid offspring, then the offspring repeatedly backcross with one of the parental species. Introgression has long been recognized as a key process in evolution, as it may contribute to speciation, diversification, and adaptation to new environments. The importance and prevalence of introgression has been well established in plant and animal systems, and in this study we use a fungal model system, Neurospora, to study the introgression at the genomic level. We gathered genomic data from six genomes, and by comparative genomics we revealed genetic transfer of DNA regions of unprecedentedly large sizes, covering over 50% of the mating-type chromosomes, and used phylogenetic analyses to reveal the origin and direction of the transfer. Introgression was found solely on the mating-type chromosomes, which contradicts theoretical predictions for sex-determining chromosomes. We argue that this unexpected pattern is due to the fact that fungi do not have differentiated sexes (female/male) and thereby are free from sex-biased evolutionary forces. Instead, we suggest that introgression between fungal species may result in reinvigoration of genomic regions exposed to suppressed recombination.

## Introduction

Introgression is a process by which two species mate and produce a hybrid offspring, after which the hybrid offspring repeatedly backcross with one of the parental species. It ultimately results in genetic material from one species infiltrating another, genetically differentiated, species [1]. Introgression is a key process in evolution, as it may contribute to speciation, diversification and adaptation to new environments [1], [2]. The importance and prevalence of introgression has been well established in plant systems. For example, Rieseberg (1993) summarized 65 cases of introgression associated with plant speciation [2]. In addition, Mallet (2005) estimated that up to 25% of plant species produce viable offspring from interspecific matings, which lead to simple hybridization and introgression [3]. In the animal kingdom, introgression has also been recognized as an important factor driving genome evolution [3]–[7], possibly transferring key genes for development among species [8], [9].

Evidence suggests that introgression does not occur randomly across genomes. For instance, introgression may be less likely to occur in genomic regions with complex hybrid incompatibility loci [1], such as sex chromosomes in heterogametic organisms (e.g., XY in mammals [10]). This phenomenon is consistent with Haldane's rule and the large X effect hypothesis, which predicts that the density of hybrid incompatibility loci will be greatest on the X chromosome in animals [11]. Introgression is also less likely to occur in regions of suppressed recombination [1], [12], [13]. Nonetheless, simulation studies have shown that if the hybrid fitness is not exceedingly low, even slightly deleterious introgressions have a good probability of fixation, due to genetic drift or population demographics [14]–[16] (see also [17]–[20]), and thus might not always be highly dependent on genomic location.

In the fungal kingdom, the role of introgression is beginning to be explored. In the past five years, interspecific gene flow, especially of virulence genes, has been found in multiple fungal systems, e.g., Coniophora, Fusarium, Microbotryum, Aspergillus, Heterobasidion and Stagnospora [21]–[27]. In these cases, introgression has provided adaptive advantages to pathogenic species. A notable pattern of introgression is also found in Neurospora, Ophiostoma and Stemphylium [28]–[30]. Reproductive genes in these systems seem to be more permeable to introgression than housekeeping genes or non-coding loci, which is in contrast to theoretical expectations [11]. Most studies of interspecific gene flow in fungi are based on multilocus data, thus being unable to reveal the actual size, abundance and distribution of introgression tracts on the genomic level. So far, only a limited number of studies have used genomic approaches to study introgression, and these studies revealed small, discrete introgression tracts in *Coccidioides posadasii* (70 introgressed regions of which 60 regions <50 kb), *Aspergillus fumigatus* (189 introgressed regions, 1 Mbp total size) and *N. crassa* (2 introgressed regions, each ∼10 kb) [31]–[33]. The growing number of genomic datasets from the fungal kingdom provides the potential to increase our understanding of introgression in fungal genomes [34]. Furthermore, the fact that fungi have no differentiated sexes, i.e., female/male dichotomy of individuals carrying gametes of different sizes, make them alternative, and simple, models to study general processes in nature expected to be affected by sex-biased evolutionary forces [35].

The filamentous ascomycete genus Neurospora is a particularly useful model system to study introgression in nature. The heterothallic (self-incompatible) and pseudohomothallic (partially self-compatible) species of Neurospora constitute the terminal clade in the genus phylogeny [36]. This clade contains several well-characterized, closely related species, which are all able to sexually outcross in nature [36]. The heterothallic species, represented by the model species *N. crassa*, grow vegetatively as haploid and require a partner with nuclei of a compatible mating type (*mat A* or *mat a*) in order to go through the sexual cycle. In contrast, the pseudohomothallic *N. tetrasperma* grows as heterokaryotic for mating type (i.e., cells harbor haploid nuclei of opposite mating types) and are thereby predominantly self-fertile. *Neurospora tetrasperma* also rarely forms homokaryotic, single mating-type individuals that undergo outcrossing events to return to the heterokaryotic stage [37], [38]. Although the species in the terminal Neurospora clade are reproductively isolated, overlapping geographical distributions may have created opportunities for hybridization between them [36], [39], [40], and indeed, fertile interspecies crosses in the laboratory have been reported [38], . Phylogenetic studies by Skupski et al. (1997) and Strandberg et al. (2010) [28], have shown different relationships between Neurospora species for genes on the autosomes and the *mat* genes, possibly reflecting introgression between species.

The pseudohomothallic *N. tetrasperma* has attracted considerable research attention. The genome of this taxon is highly syntenic to the model species *N. crassa*, which harbors 7 chromosomes in 41 Mbp (http://www.broadinstitute.org). However its mating-type (*mat*) chromosomes contain a large region of suppressed recombination (75% of the chromosome, or >5 Mbp) [35], [37], [43], [44]. Consequently, *N. tetrasperma* provides a model system to study the evolution of sex chromosomes [35]. The suppressed recombination region evolved 3.5–5.8 MYA ago [35]; this is in a similar range to that of other young sex chromosomes (e.g., Silene [45]–[48]). A recent analysis of high quality genomic data from two *N. tetrasperma* homokaryotic strains (FGSC 2508 and FGSC 2509) of opposite mating type revealed that the *mat A* chromosome has experienced a history of three inversions that encompasses the majority of the region of suppressed recombination, while the *mat a* chromosome is collinear with the *N. crassa mat* chromosome [49]. Recent molecular evolution studies have revealed genetic degeneration in the region of suppressed recombination in *N. tetrasperma*, as evidenced by reduced codon usage bias and the accumulation of non-synonymous substitutions, as compared to the flanking, recombining, regions of the chromosomes [49]–[51]. These data are consistent with genomic degeneration in the young regions of suppressed recombination, similar to trends reported for ancient eukaryotic sex chromosomes [45], [48], [52]–[54]. Notably, asymmetrical degeneration between the two *mat* chromosomes has been found [49], [51], which was suggested by Whittle et al. (2011) [51] to be caused by factors such as chromosome-specific structural rearrangements on the *mat A* chromosome [44], [49] and/or rare outcrossing or interspecific hybridization events [38], [55]. *Neurospora tetrasperma* was first described by morphological characters [56], and several studies have indicated that the pseudohomothallic mating system of *N. tetrasperma* is derived from true heterothallism, and that it is monophyletic [38], [42], [57]–[59]. Furthermore, *N. tetrasperma* has recently been recognized as a species complex consisting of multiple genetically and largely reproductively isolated lineages, for which the relationship is largely unresolved [38].

The aim of the present study was to use comparative genomics of Neurospora species to investigate the introgression landscape at the genomic level. We acquired medium coverage genomic data from multiple *N. tetrasperma* lineages, and by using interspecific genomic comparisons with *N. crassa* we revealed the abundance, size and distribution of introgression tracts among the species. We used multilocus genealogies from additional heterothallic species of Neurospora to infer the direction of introgression events. Finally, we investigated patterns of molecular evolution in the introgressed regions.

## Results

### Draft genome sequences

We sequenced the *mat A* and *mat a* haploid genomes from three wild-type heterokaryotic strains of *N. tetrasperma*, selected to represent three genetically and reproductively isolated lineages [38]. The six haploid genomes are referred to herein by their lineage ID followed by mating type, i.e., L1A, L1a, L4A, L4a, L9A and L9a (Table S1). Approximately 7 million illumina paired-end reads were generated and mapped to the reference genome of *N. crassa* (release version 10, ∼41 MB), yielding an ∼15-fold medium coverage data for each of the six haploid genomes (Table S2). Reads covered ∼80% of the genomes, and were evenly distributed (coverage depth 10–20X), except repeat-enriched centromeric regions (coverage depth <5X) (Table S2, Figure S1). The seven assembled chromosomes are referred to as linkage group I (LGI, also referred to as the *mat* chromosome), LGII, LGIII, LGIV, LGV, LGVI, LGVII, as in *N. crassa* [60].

### Reference phylogeny

**Figure 1:**
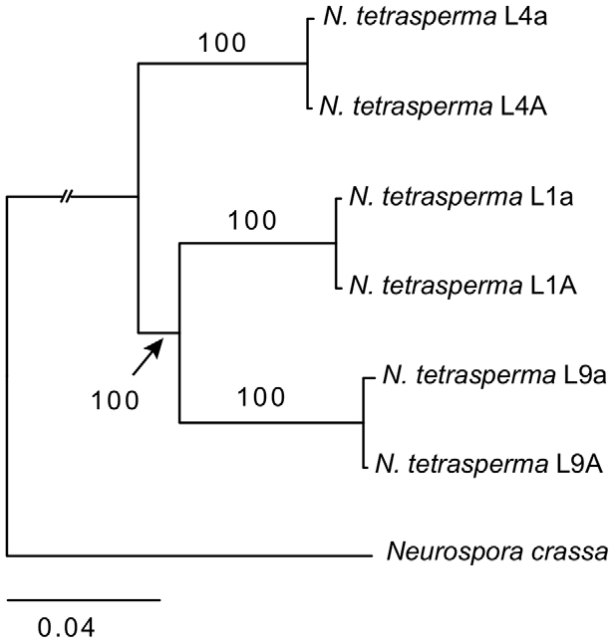
Phylogenetic relationship of the investigated lineages of *N. tetrasperma*. The tree was generated by maximum likelihood analysis of 1,978 concatenated autosomal genes. Bootstrap value from 100 replicates is shown by each branch.

We inferred the phylogenetic relationship of our selected *N. tetrasperma* lineages and *N. crassa*, using a concatenated dataset of 1,978 autosomal genes with the maximum likelihood method. The tree topology (Figure 1) shows that lineage 4 (RLM131) represents a slightly earlier diverging lineage than lineage 1 (P4492) and lineage 9 (965). Importantly, for the purpose of this discussion, the autosomal alleles of the two strains of different mating types from each lineage (e.g., L9A and L9a) were in all cases nearly identical.

### High levels of intra-lineage sequence divergence in *mat* chromosomes of *N. tetrasperma*

We investigated the sequence divergence between pairs of chromosomes of the same lineage of *N. tetrasperma*, and found strikingly different patterns for the autosomes and the *mat* chromosome. The overall chromosomal divergence levels between the haploid genomes ranged from 3.7×10^−3^ to 5.5×10^−3^ substitutions per site for the six autosomes (Table S3), which is 2–4 fold lower than the overall divergence between the *mat* chromosomes, for which we found 1.0×10^−2^ to 1.6×10^−2^ substitutions per site (Table S3). To study the variation in sequence divergence across the chromosomes for haploid genomes within lineages, we used a sliding window approach, with a window size of 500 kb and a step size of 100 kb. For the autosomes, divergence levels were relatively homogenous across the chromosomes, with the exception of slight increases at the flanking left ends of LGII, III, IV and V, at the flanking right end for autosome VI and in the central segment of autosome VII (Figure S2A). However, even at the peak, the overall levels of divergence did not exceed 1% in any of the autosomes. The regions of increased divergence also showed lower G+C content levels; 44–48%, as compared to more than 50% in all other regions of the autosomes (Figure S2C). Previous studies of LGI, LGIII and LGVII in *N. crassa* [61], [62] suggest that these sites correspond to the centromeres.

**Figure 2:**
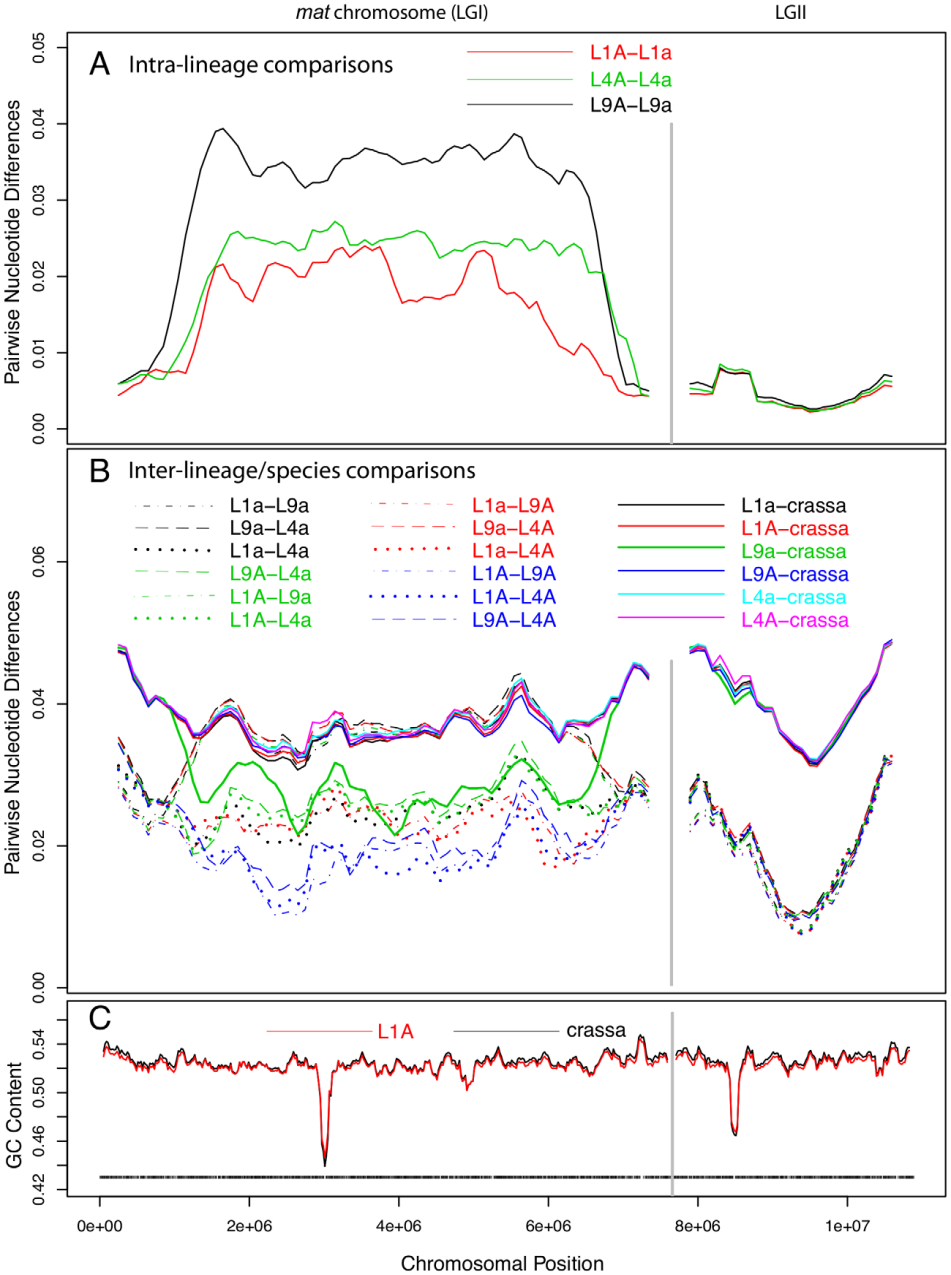
Pair-wise divergences of the mating-type (*mat*) chromosomes and one selected autosome of *Neurospora tetrasperma* lineages and *Neurospora crassa*. The pair-wise divergences were calculated as the fraction of differences (in bp) between the sequences, using a sliding window over the seven-way alignment. Lines represent 500 kb window size (step size 100 kb) sliding along the chromosome. The divergences are shown for the *mat* chromosome (LGI, left) and one of the autosomes (LGII, right). A: Intra-lineage comparisons (*mat A* vs *mat a*) of the three investigated lineages of *N. tetrasperma*: L1, L4 and L9. B: Comparisons of genomes from different lineages of *N. tetrasperma* (inter-lineage comparisons, dashed lines), and of *N. tetrasperma* and *N. crassa* (inter-species comparisons, solid lines). Note that the *mat A* – *mat A* inter-lineage comparisons (L1A–L4A, L1A–L9A and L4A–L9A) are plotted with blue lines. C: GC content in *N. crassa* (black line) and *N. tetrasperma* L1 (red line). Window size is 100 kb, step size 20 kb. Black bars indicate gene density in L1A.

The sequence divergence profile of the *mat* chromosomes was markedly different from those of the autosomes in that the central portion showed 5 to 10-fold higher divergence levels (Figure 2A). As in the autosomes, we recorded a short region of lower G+C level for the *mat* chromosomes (Figure 2C), presumably at the site of the centromere. However, the segment showing increased divergence levels was much larger, suggesting that this pattern is not explained by the location of centromeres. On the right and left flanks of the *mat* chromosomes, the intra-lineage divergence levels dropped to less than 1% (Figure 2A), which is a similar level as observed for the autosomes (Figure 2, Figure S2A); these flanking regions of the *mat* chromosomes are referred to as pseudoautosomal (PA) regions. The size of the region with divergence levels above 1% was estimated to be at least 5.37, 5.86 and 5.88 Mb in lineages L1, L4 and L9, respectively (Table S4), covering about 70–77% of the chromosome size. The overall divergence level of the central part of the *mat* chromosome differed between each lineage (Figure 2A, Table S4, L1: 0.0187, L4: 0.0238, L9: 0.0343), as did the border positions to the pseudoautosomal (PA) regions (Figure 2A, Table S4), suggesting that the *mat* chromosomes have independent evolutionary histories in the three lineages.

### Introgression of foreign DNA into the *mat a* chromosomes of *N. tetrasperma*

Atypically high nucleotide sequence divergence levels between the *mat* chromosomes within a lineage could be due to transfer of DNA from another species (i.e., introgression) or to reduced rates of sequence exchange across the two *mat* chromosomes (i.e., suppressed recombination). In the first case, we expect one of the two chromosomes to show a closer relationship to a *mat* chromosome of another species, e.g. *N. crassa*, than to its homolog of the same lineage. However, under the second hypothesis, we expect the two homologous chromosomes of each lineage to show the same divergence to other species. Furthermore, reduced sequence exchange by suppressed recombination is expected to lead to the same divergence between the *mat A* and the *mat a* chromosomes of *N. tetrasperma*, i.e., the *mat A* chromosomes should be as similar to each other as the *mat a* chromosomes. To test these hypotheses, we examined the level of sequence similarity of each *N. tetrasperma* genome to all other *N. tetrasperma* genomes included herein, and to the outgroup species *N. crassa*.

For the autosomes, as expected, the nucleotide divergences of the *N. tetrasperma* haploid genomes of the same lineage (Figure S2A, Table S3; 0.0045±0.0006) were considerably lower than divergences obtained by inter-lineage comparisons among genomes of *N. tetrasperma* (dotted and dashed lines of Figure S2B, Table S3; 0.0208±0.0016), which, in turn, were lower than inter-species divergences of *N. tetrasperma* and *N. crassa* genomes (solid lines of Figure S2B, Table S3; 0.0423±0.00054) (Table S3). In support of introgression, the pattern of divergence obtained from the central region of the *mat a* chromosomes stands in striking contrast to that of the autosomes. In L9, comparison of sequence divergence in the central region of the *mat* chromosome showed a lower level of divergence when the L9 *mat a* strain of *N. tetrasperma* (i.e., L9a) was compared to *N. crassa* than when L9a was compared to L9A (0.027 vs 0.034; Figure 2, Tables S4, S5). This difference in divergence was supported by a shared-nucleotide test [6], which revealed a significantly higher number of uniquely shared nucleotides between L9a and *N. crassa* than between L9A and *N. crassa* in this region (122,510 vs. 74,478, Binomial Sign Test P<10^−10^; nt identity, L9a-*N. crassa* 97.3%, L9A-*N. crassa* 96.4%). This pattern was not found for any other of the *N. tetrasperma* sequences (L1, L4) in comparison with *N. crassa*. However, in support for introgression of all lineages, the pattern of divergence of the *mat A* chromosomes stands in contrast to that of the *mat a* chromosomes. Specifically, the divergence levels of all three pair-wise comparisons among the *mat A* chromosomes were lower than the divergences between the *mat a* chromosomes of L1, L4 and L9 (L1A–L9A: 0.0169, L1A–L4A: 0.0166, L4A–L9A: 0.0183, L1a–L9a: 0.0360, L1a–L4a: 0.0240, L4a–L9a: 0.0370). This difference in divergence was supported by a shared-nucleotide test, which revealed that the *mat A*-*mat A* chromosomes shared more unique nucleotides than *mat a*-*mat a* in all pair-wise comparisons (for example, L9 mat A = L4 mat A≠L4 mat a 82,774 vs. L9 mat a = L4 mat a≠L4 mat A 61,973, P<1×10^−10^, Binomial Sign Test). Taken together, these results indicate infiltration of foreign DNA, i.e., introgression, into the *mat a* chromosomes of all investigated lineages of *N. tetrasperma*.

### The size of the introgression tract

**Figure 3:**
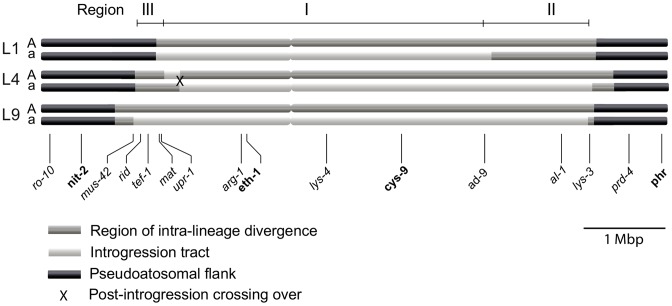
Schematic illustration of the mating-type (*mat*) chromosomes of the three *N. tetrasperma* lineages included in the study. Regions of elevated intra-lineage divergence and introgression are defined based on genome divergence estimates. Genes positioned below the chromosomes were used for the phylogenetic and Bayesian concordance analyses. In bold are genes for which representatives of all known heterothallic species and subgroups of Neurospora were included in the phylogenetic analyses. The figure is drawn to scale.

We determined the size of the introgression tracts of the *mat a* chromosomes as follows. In L9 we estimated the tract to be 5.6 Mbp (73% of the *mat a* chromosome) by nucleotide difference comparison of L9A-*N.crassa* and L9a-*N. crassa*, in L1 to 4.1 Mbp (53% of the *mat a* chromosome) by comparing L1A–L9A and L1a–L9A, and in L4 to 5.2 Mbp (68% of the *mat a* chromosome) by comparing L4A–L9A and L4a–L9A. We chose *N. crassa* or L9A as reference strains based on the divergence patterns of Figure 2B. The estimated borders of the introgression tracts are shown in Table S4, and schematically depicted in Figure 3.

For each lineage, the introgression tract was confined to the region of elevated divergence (Figure 3, Table S4). In the *mat a* chromosomes of L9 and L4 the introgression tracts extended the majority of the divergent region, while in L1, the introgression tract is 1.28 Mbp shorter than the region of elevated divergence (Figure 3, Table S4). The right border of the introgression tract is shared between L9 and L4, while the left border differs slightly for all lineages (Figure 3, Table S4). We refer to the region that is introgressed in the *mat a* chromosomes of all three heterokaryotic lineages as Region I and the right flank region introgressed in L4 and L9, but not in L1, as Region II. The region on the left flank, which shows a variable pattern of introgression and divergence among the three lineages, is referred to as Region III (Figure 3).

### Single gene trees establish the origin and direction of introgression

**Figure 4:**
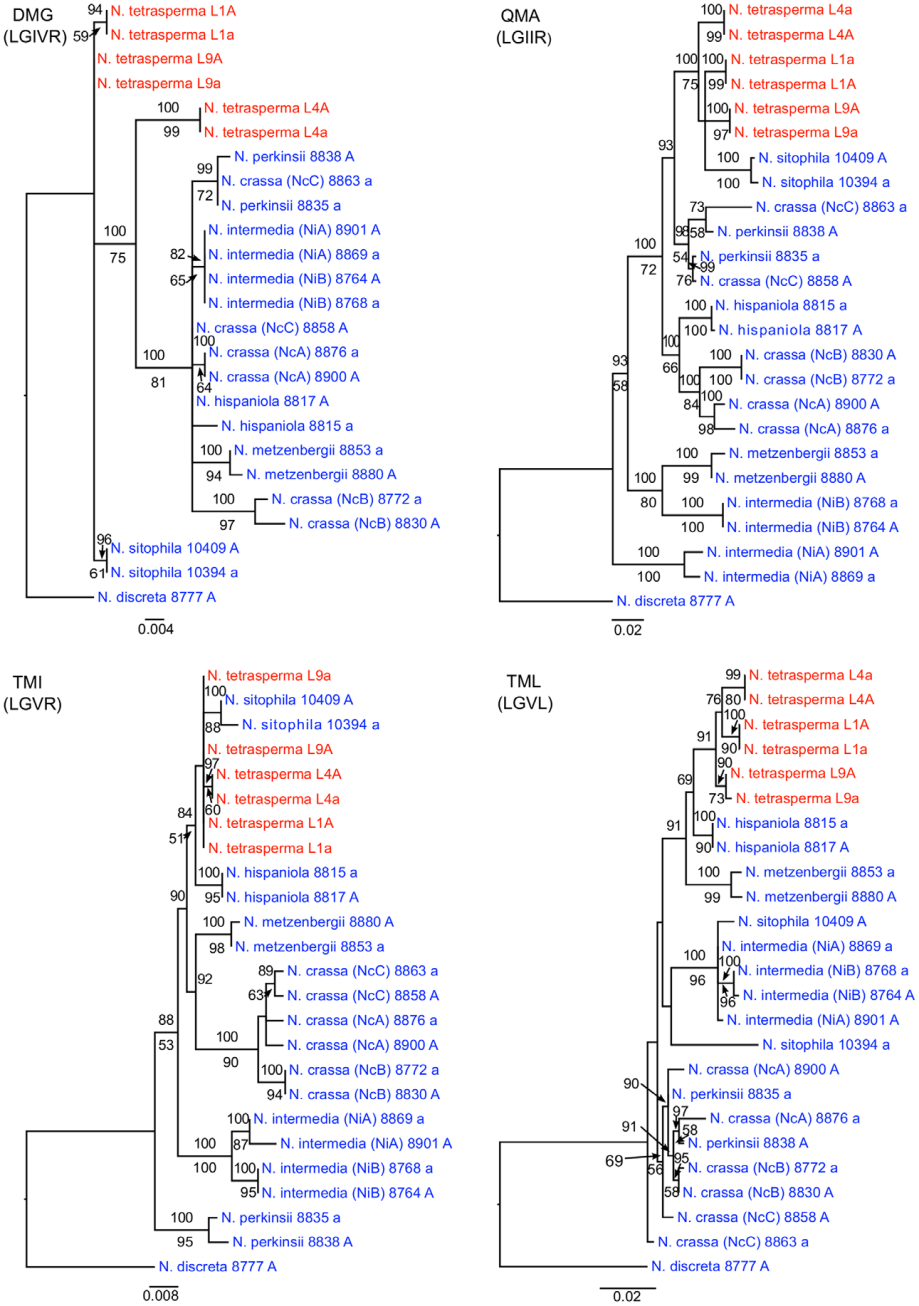
Phylogenies reconstructed for four microsatellite flanking loci (DMG, QMA, TMI, and TML) located on the autosomes of Neurospora. Each tree includes representatives of three *N. tetrasperma* lineages (red), and ten heterothallic Neurospora species (blue). *Neurospora discreta* was used as outgroup in all analyses. Chromosomal location (linkage group, right or left flank) is given within parenthesis by each locus name. The topologies shown are from the Maximum likelihood phylogenetic analyses, with the bootstrap supports for the analysis shown below the branches and the posterior probabilities from the Bayesian phylogenetic analysis shown above the branches.

**Figure 5:**
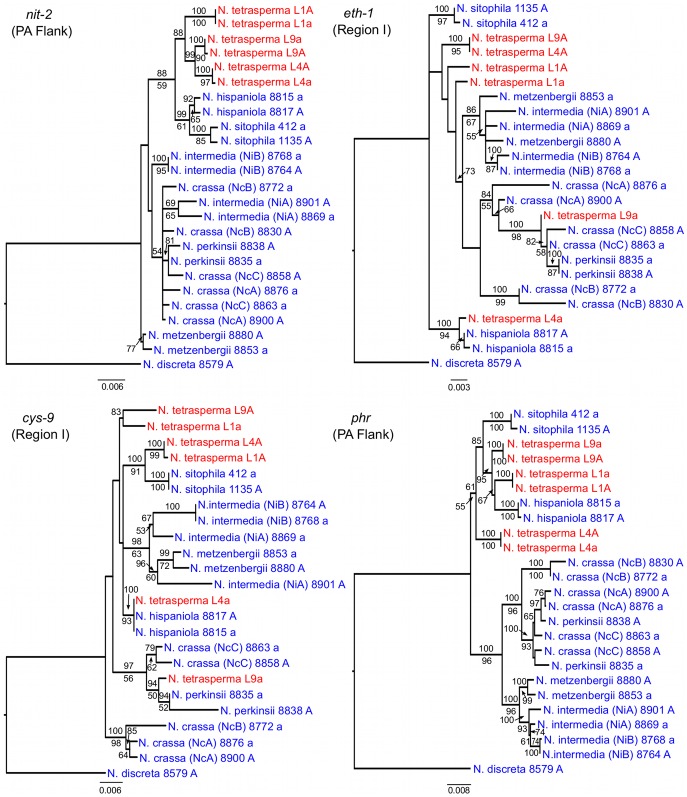
Gene genealogies for *nit-2*, *eth-1*, *cys-9*, and *phr* located on the mating-type (*mat*) chromosome of Neurospora. The genes *nit-2* and *phr* are located in the left and right pseudoautosomal (PA) flank, respectively. The genes *eth-1* and *cys-9* are located in the central region of the chromosome, for which the *mat a* chromosomes of all lineages of *N. tetrasperma* (L1a, L4a and L9a) show indications of introgression. Each genealogy includes representatives of the three investigated *N. tetrasperma* lineages (red), and ten heterothallic Neurospora species (blue). *Neurospora discreta* was used as outgroup in all analyses. The topologies shown are from the Maximum likelihood phylogenetic analysis with the bootstrap supports for the analysis shown below the branches and the posterior probabilities from the Bayesian phylogenetic analysis shown above the branches.

To identify the origin and direction of introgression, we carried out phylogenetic inferences of lineage relationships for single loci located on the autosomes and the *mat* chromosome (10 autosomal loci and 16 loci on the *mat* chromosomes, Figure 3, Table S6). For four microsatellite flanking loci located on autosomes (Table S6) and four gene loci evenly distributed across the *mat* chromosomes (highlighted in bold in Figure 3), we included in the analyses all currently identified heterothallic species and subgroups of Neurospora (Table S1, trees shown in Figure 4), while for the additional loci a subset of heterothallic species were included (Table S6, trees shown in Figure S3). The results revealed that for all autosomal genes, the two haploid single mating-type components of each lineage clustered together (Figure 4, Figure S3), as also observed in the tree topology from concatenated genes of the autosomes (Figure 1). Likewise, the genes located on the PA flanking ends of the *mat* chromosomes show near identity in comparisons of alleles from *mat A* and *mat a* haploid genomes (trees of *nit-1* and *phr* are shown in Figure 5, and *ro-10*, *mus-42*, *prd-4* in Figure S4), consistent with homogenization by recombination.

In contrast, phylogenies inferred from genes located in the central region of the *mat* chromosomes showed no clustering of the *mat A* and *mat a* chromosomes from the same lineage (Figure 5). For both of *eth-1* and *cys-9*, L9a clustered with *N. crassa* subgroup C and *N. perkinsii* (bootstrap support 84-97%), and the sequence similarity between L9a and *N. crassa* (NcC, strain 8863) and *N. perkinsii* (strain 8835) is 99.2% and 99.6%, respectively, for these two genes. L4a clustered with *N. hispaniola* (100% bootstrap support), and showed a very high sequence similarity with strain 8817 (99.8%). These patterns were supported by the additional trees of genes from the *mat* chromosome, for which only a subset of the heterothallic taxa were included (Figure S4). Note that in L4, for genes between *rid-1* and *upr-1* (i.e., region III, Figure S4) the alleles from the *mat A* chromosome (and not the *mat a* chromosome) clustered together with *N. hispaniola*. A possible explanation for this observed discord is the occurrence of a relatively recent crossover event between the *mat* chromosomes in L4, at a chromosomal location between *upr-1* and *arg- 1*. Indeed, evidence for a crossover on the *mat* chromosome at this location for L4 has previously been shown [63]. Furthermore, for the genes of region III, in which the divergence indicate no introgression of L4a (Figure 3), gene tree analyses support introgression from *N. hispaniola*, indicating that the divergence method is likely to underestimate the size of the introgression tract. Finally, in the gene *lys-4*, both alleles of L4 cluster together, consistent with a gene conversion event reported for this gene in this lineage [63].

### Bayesian concordance analyses support introgression

**Figure 6:**
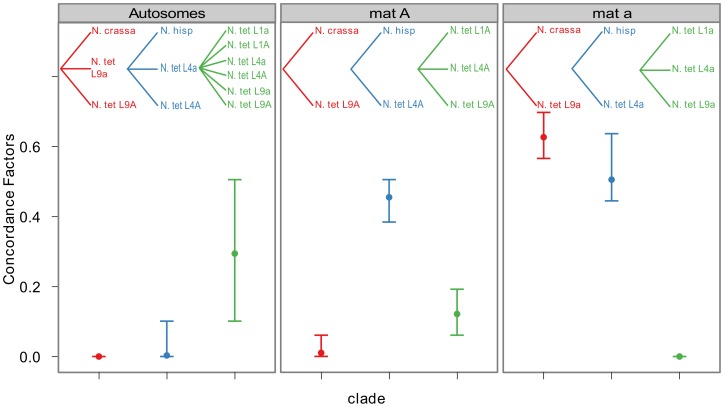
Bayesian concordance factors of selected clades, for the autosomes and the mating-type chromosomes (*mat A* and *mat a*) of Neurospora. Bayesian concordance factors and 95% credibility intervals (error bars) estimated for 10 loci on the autosomes and 16 loci on the *mat* chromosomes. Concordance factors are given for the three hypotheses illustrated as clades in the plot, red: *N. tetrasperma* L9 sister species to *N. crassa*; blue: *N. tetrasperma* L4 sister species to *N. hispaniola*; green: *N. tetrasperma* monophyletic. The results displayed were calculated with the prior alpha set to 1.

To quantify the amount of gene tree discordance for the different genomic regions, we performed a Bayesian concordance analysis (BCA) of the 26 loci of Table S6, using the program BUCKy v1.4.0 [64]. A BCA analysis infers concordance factors as a measure of the proportion of loci that support a given bipartition of the data [65]. Here we employed the BCA to see if we observe a different inferred history from autosomal loci versus *mat* chromosome loci, as such a non-random pattern may be due to the action of introgression rather than incomplete lineage sorting. The analysis was performed using all included lineages of *N. tetrasperma*, and representatives of the four heterothallic species *N. crassa*, *N. sitophila*, *N. hispaniola* and *N. discreta* (Table S1, Table S6). In Table S7 and Table S8 we show the sample-wide concordance factors (CF) and 95% credible intervals for clades in the analysis of autosomal and *mat* chromosome loci, respectively, and in Figure 6 we present a plot of the sample-wide CFs for three conflicting clades of specific interest: the L9-*N. crassa* clade (red), the L4*-N. hispaniola* clade (blue) and the *N. tetrasperma* clade (a monophyletic grouping of all six *N. tetrasperma* lineages; green) in different genomic regions. We found that the estimate of CF is robust to the change of the prior alpha (a test of the effect of changing the prior is shown in Figure S5) and the results presented here are from the analysis with the prior set to 1.

For the autosomal loci, the dominant history observed is the *N. tetrasperma* clade with a CF of 0.291 (95% credible interval 0.1–0.5), while the L9-*N. crassa* clade and the L4-*N. hispaniola* clade received little or no support (CF<0.0001 for both clades: Figure 6, Table S7, Figure S6). Other clades that conflicted with the monophyly of *N. tetrasperma* (Table S7) showed lower CF values; however, their 95% credibility intervals did overlap with the *N. tetrasperma* clade (e.g., 1,2,5,7,8|3,4,6,9,10: Table S7). This overlap means that for the autosomes we cannot statistically designate the *N. tetrasperma* monophyly as being the dominant history for the *N. tetrasperma* lineages, although the CF is highest for this clade when compared to those clades that are in conflict with it.

**Figure 7:**
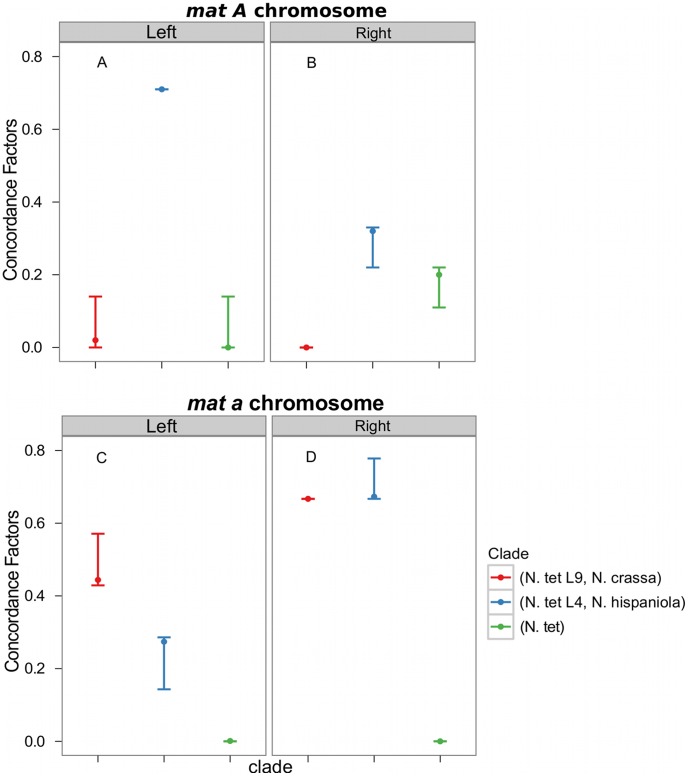
Concordance factors on the mating-type (*mat*) chromosome support a post-introgression crossover between the *Neurospora tetrasperma* L4 *mat* chromosomes. The concordance factors are plotted to the left and right of the crossover event described by Menkis et al. (2010) for both the *mat A* (A and B) and *mat a* (C and D) chromosomes. Concordance factors are given for the three hypotheses illustrated as clades in the plot, red: *N. tetrasperma* L9 sister species to *N. crassa*; blue: *N. tetrasperma* L4 sister species to *N. hispaniola*; green: *N. tetrasperma* monophyletic. The results displayed were calculated with the prior alpha set to 1.

Consistent with introgression of the *mat a* chromosome of the *N. tetrasperma* L9 from *N. crassa*, we observed a high CF on the *mat a* chromosome for the L9a-*N. crassa* clade (CF 0.616, 95% credible interval 0.562–0.688), while no support for this clade on the *mat A* chromosome or autosomes was found (Figure 6). This pattern is less apparent for the L4-*N. hispaniola* clade. The CF for this clade is relatively high for both *mat* chromosomes, i.e., it is noticeably higher than expected for the *mat A* chromosome (0.453 (0.375–0.5)): Figure 6, Table S8). A possible explanation for this observed discord is the crossover event between the *mat* chromosomes in L4 previously reported by Menkis et al. (2010) [63]. To further investigate the transfer of genetic material between *mat* chromosomes of L4 as the reason for the discordance, we partitioned our data into loci that are to the left and right of the location of the crossover event (proposed by Menkis et al. (2010) to be between markers *upr-1* and *arg-1* (Figure 3) [63]), and carried out BCA analyses of the two separate datasets. The plots of the CFs to the opposite sides of the inferred crossover event support the view that a crossover event has transferred a part of the introgressed region from the *mat a* to the *mat A* chromosome; the CF for the RLM131-*N. hispaniola* clade is higher on the left of the crossover on the *mat A* and lower to the right, while the opposite pattern is observed for the *mat a* chromosome (Figure 7).

Taken together, the results show that the pattern of phylogenetic discordance on the *mat a* chromosome is distinct from the patterns observed for the *mat A* chromosome and the autosomes, and provides further support for large-scale introgression tracts on the *mat a* chromosome of the *N. tetrasperma* lineages under study.

### Introgression is associated with reduced molecular degeneration

In order to reveal whether the previously reported signs of asymmetrical degeneration between the two *mat* chromosomes in *N. tetrasperma* [49], [51] can be explained by introgression of large chromosomal regions from freely recombining heterothallic species, we analyzed substitution frequencies at nonsynonymous and synonymous sites, and codon usage patterns, in a concatenated data set of complete coding sequences (CDS) for 543 genes from the region of the *mat* chromosomes subjected to introgression in all investigated lineages of *N. tetrasperma* (Region I, Figure 3). We expected the degeneration to be reduced in introgressed regions, and followed the method developed previously for studying molecular degeneration in *N. tetrasperma*, for which an increase in ω (dN/dS) was verified to be due to the accumulation of slightly deleterious mutations and that the accumulation of non-preferred codons were not due to a mutational bias [50], [51].

Consistent with the expectations, an elevated ω on *mat A* chromosomes, as compared to the *mat a* chromosomes, was revealed by using branch models of ω implemented in the codeml program of the Phylogenetic Analysis by Maximum Likelihood (PAML) package [66] on the concatenated gene sequence data. First, we estimated ω for each branch in the phylogeny by running the free ratio model. The result from this test indicated that the three branches delineating the *mat A* chromosomes have higher ω (L9A: ω = 0.2005; L1A: ω = 0.1937; L4A: ω = 0.1976) than the *mat a* branches (L9a: ω = 0.1450; L1a: ω = 0.1696; L4a: ω = 0.1562). The ω value for the branch of the freely recombining heterothallic species, *N. crassa*, was 0.1229, which was the lowest among all seven branches in the input phylogeny. Furthermore, within each lineage, we found a significantly better fit for a local model of ω allowing a different ω on *mat A* chromosomes than *mat a* chromosomes (P<1×10^−10^ for L9, P = 3.7×10^−3^ for L1, P = 1.5×10^−8^ for L4: in each of these cases ω was higher in the *mat A* chromosomes). Finally, a local branch model in which the three branches delineating *mat A* chromosomes and the three *mat a* branches were allowed to have a separate ω, fitted the data significantly better than the global model (P<1×10^−10^, *mat A* branches ω = 0.1853, *mat a* branches ω = 0.1474).

We assessed the accumulation of non-preferred (NPR) codons relative to preferred (PR) codons in the 543 genes for all six haploid genomes, to test if asymmetrical degeneration can be detected on the level of synonymous codon substitution. We found a net accumulation of NPR relative to PR in all six *N. tetrasperma* haploid genomes (Binomial Sign test, p<1×10^−10^) (Table 1). Also, we report statistically significantly higher number of NPR substitutions (than PR) in L1A and L4A as compared to L1a and L4a (Binomial Sign test, P = 1.6×10^−10^ for L4, P = 7.1×10^−3^ for L1), while the test was inconclusive for L9A/L9a.

**Table 1:** The number of synonymous allele specific changes from preferred codons (PR) to non-preferred (NPR) codons and from NPR to PR in the introgressed region of *N. tetrasperma* Lineage 1, Lineage 4, and Lineage 9, relative to *N. crassa*.

*mat* chromosome (Type of Codon Switch)	Type of codon located in:			No. of switches	No. of excess switches of PR to NPR^1^	Frequency of excess switches of PR to NPR per 1000 codons^2^
	NC	*mat a*	*mat A*			
**Lineage 1**						
*mat a* (PR to NPR)	PR	NPR	PR	1308	510	1.80
*mat a* (NPR to PR)	NPR	PR	NPR	798		
*mat A* (PR to NPR)	PR	PR	NPR	1531	736	2.60
*mat A* (NPR to PR)	NPR	NPR	PR	795		
**Lineage 4**						
*mat a* (PR to NPR)	PR	NPR	PR	1828	765	2.70
*mat a* (NPR to PR)	NPR	PR	NPR	1063		
*mat A*(PR to NPR)	PR	PR	NPR	1827	875	3.09
*mat A* (NPR to PR)	NPR	NPR	PR	952		
**Lineage 9**						
*mat a* (PR to NPR)	PR	NPR	PR	2089	812	2.87
*mat a* (NPR to PR)	NPR	PR	NPR	1277		
*mat A*(PR to NPR)	PR	PR	NPR	3300	751	2.66
*mat A* (NPR to PR)	NPR	NPR	PR	2549		

1 No of excess switches = No of switches from PR to NPR – No of switches from NPR to PR. It represents the allele specific excess of non-preferred codon changes.

2 Frequency of excess PR to NPR switches per 1000 = (No of excess of switches/No of total codons)×1000. The total codons for Lineage 1, Lineage 4 and Lineage 9 are 283392, 282819 and 282723, respectively. The number differs due to the removal of non-synonymous change codons.

Taken together, by using the methods previously developed for studying molecular degeneration associated with suppressed recombination [50], [51], we found an elevated genomic degeneration in the *mat A* chromosomes, which have not been subjected to introgression.

## Discussion

The combination of comparative genomics and phylogenetic methods is a useful approach to study introgression between species. In particular, variable divergence patterns between genomes may provide evidence of the location and size of introgression tracts [6], [31], and gene tree analyses may give clues toward the origin and direction of introgression [67], [68]. Furthermore, statistical analysis of gene tree concordances allow for an evaluation of the different biological processes affecting genome evolution, such as hybridization, introgression and incomplete lineage sorting [69], [70]. Using these methods, we detected large-scale introgression in the non-recombining region of the *mat a* chromosome in all three investigated *N. tetrasperma* lineages (covering >50% of the *mat* chromosome and at least 4 Mbp), and show that the introgression likely originates from two closely related, freely recombining, heterothallic species: for genes on the *mat a* chromosome of L4 and L9, we found an almost complete match with *N. hispaniola* and *N. crassa NcC*/*N. perkinsii*, respectively. In addition, we report that the introgression is associated with reduced genomic degeneration in *mat a* chromosomes, consistent with a shortened time period of recombination suppression. Notably, the chromosome specific (*mat*) and allele specific (*mat a*) introgression reported herein comprise, to our knowledge, the largest introgression tracts reported from natural populations to date [3], [28]–[31], [33], [71]. Furthermore, our data contradicts current notions that introgression should be less likely on chromosomes determining sexual identity [1], [72].

The unique divergence patterns of large fractions of the *mat a* chromosomes of the *N. tetrasperma* lineages in this study (Figure 2B) provide strong indications of introgression, and this result is supported by the Bayesian concordance analyses (BCA). While BCA makes no assumptions on the causes of discordance, it has been proposed that examination of concordance factors for conflicting clades of interest may be used to reject incomplete lineage sorting as the cause of discordance [69]. In this study, the discordance pattern on the *mat a* chromosome shows a strikingly different pattern to the pattern observed on the *mat A* chromosome and the autosomes. A history of introgression along the *mat a* chromosome would be expected to increase the support for the clades composed of the heterothallics and the introgressed *N. tetrasperma* lineages. We would also expect that the monophyly of *N. tetrasperma* would receive lower support (CF) in introgressed regions. Both of these expectations fit with our results from the BCA analysis (Figure 6). An alternative explanation for the patterns observed in this study is the action of balancing selection. However, we do not expect to see patterns of deviating divergence estimates extending over large physical distances of the *mat* chromosome between *N. tetrasperma* lineages and the freely recombining *N. crassa*, if balancing selection is the sole driving evolutionary force. Specifically, our consistent observation of a high sequence similarity between L1a and *N. hispaniola*, and L9a and *N. crassa NcC/N. perkinsii*, for multiple genes across the *mat* chromosome, indicates that the observed patterns are more likely to be the result of introgression between species.

### Large introgression tracts on fungal mating-type chromosomes

Sex chromosomes, such as the XY in mammals and ZW in birds, and *mat* chromosome in *N. tetrasperma* are similar in two main aspects. Firstly, both harbor a large region of suppressed recombination, which has expanded in a punctuated manner into evolutionary strata [35], [73], [74]. Secondly, both have experienced genomic differentiation and degeneration after the cessation of recombination [50], [51], [74]. However, in terms of introgression, the two sex-determining systems apparently show variable features. Introgression is unlikely to fixate in most sex chromosomes, since the density of hybrid sterility genes are much higher in sex chromosomes than autosomes, as predicted by Haldane's rule and the large X-effect [11]. Empirical support for this expectation comes from a wide range of animal taxa including mammals, Drosophila, birds and butterflies [75]–[77]. However, in the fungal kingdom, empirical data have shown mixed signals. Turner et al. (2011) reported that a large fraction of QTLs for reinforcement of reproductive barriers between heterothallic Neurospora are found on the *mat* chromosome [78]. Nevertheless, three previous genealogy studies have found introgression to be enriched in *mat* chromosome loci compared to autosomal loci [28]–[30]. These previous findings together with the large and continuous introgression tracts (covering >50% of *mat* chromosome) reported in this study are suggestive of a relationship between introgression and mating-type loci/chromosomes in fungal species. The deviation from Haldane's rule and the Large X-effect may originate from the system of determining sexual identity in fungi. For example, the causes of hybrid sterility in sex chromosomes of animals and plants, including dominance theory, faster-male evolution, faster-X evolution and sex ratio meiotic drive, may not apply to fungal species, which typically exhibit a lack of asymmetry at the mating-type chromosomes and a mixed asexual/hermaphroditic sexual life cycle. In the absence of the above-mentioned negative effects of introgression occurring on the mating-type loci/chromosomes in fungi, other factors such as genetic invigoration could be the driving forces for the observed association (see next section).

### Evolutionary significance of introgression in *N. tetrasperma*

In previous studies we have demonstrated a genetic degeneration in the region of suppressed recombination of the *mat* chromosomes of *N. tetrasperma*, both by the accumulation of non-synonymous substitutions and non-preferred synonymous codons [50], [51], which fits with theoretical expectations for a genomic region with suppressed recombination [54]. Notably, Whittle et al. (2011) reported an asymmetry of degeneration for the two *mat* chromosomes of *N. tetrasperma* strain P4492L1 [51] and such an asymmetry was later reported by Ellison et al. (2011) [33] also for strain P581, belonging to lineage 6 (L6 [38]: not included in this study, see *Alignment and divergence estimation* in materials and methods section). While Whittle et al. (2011) found an elevated degeneration of *mat A* in L1, Ellison et al. (2011) found an elevated degeneration in *mat a* of L6 [49]–[51]. Alternative explanations for an asymmetric degeneration between the *mat* chromosomes of *N. tetrasperma* may be proposed, such as uneven level of haploid selection in the two nuclei during the heterokaryotic life cycle [79], [80], or introgression. Here, we confirmed by analyses of both dN/dS and codon usage that the *mat A* chromosome of L1 (as well as L4 and L9) has experienced more degeneration than the *mat a* chromosome, and we were able to correlate degeneration to the process of introgression. Specifically, we hypothesize that the observed introgression of *mat a* chromosomes in *N. tetrasperma* from species with free recombination is adaptive, by reducing degeneration levels in this chromosome.

An alternative, neutral, hypothesis is that introgression into *N. tetrasperma mat* chromosomes is a result of genetic drift. Currat et al. (2008) demonstrated through simulations that during a range expansion process, if the hybrid fitness is not too low, the genome of invading species should be massively introgressed by local species [15], [16]. Consequently, a pattern of unidirectional introgression as observed here could be explained by the range expansions of different selfing *N. tetrasperma* populations. The patterns of the phylogenies on the *mat* chromosomal loci (*cys-9* and *eth-1*) from within the introgression tract support a history of unidirectional introgression from the heterothallic species into the *N. tetrasperma* populations. This may be consistent with a past expansion of the self-fertile *N. tetrasperma* populations into locations where heterothallic species were already present. Such invasions may be due to a fitness advantage of *N. tetrasperma* being able to self-fertilize, being functionally diploid and/or having a broader niche space. The introgression would then predominantly be from the resident heterothallic species into the invading *N. tetrasperma* population. Such a pattern has been described in a number of other taxa and has been demonstrated in simulation studies [15]. Additionally, a high degree of selfing, as expected for *N. tetrasperma* would be expected to reduce the effective recombination rate and thereby act to slow the purging of maladapted introgressed regions, in comparison to its outcrossing counterparts [81], [82]. Future studies with higher genomic coverage and/or population genomic data may reveal smaller tracts of introgression also on the autosomes, e.g., [33], but the contrasting pattern between the two chromosome types of *N. tetrasperma* presented herein would most likely stay apparent. Taken together, while the causes of introgression may be due to genetic drift or demographic process, our results indicate that the consequences of introgression may bring adaptive advantages for *N. tetrasperma*. In the future, consequences of introgression, such as fitness estimates, can be studied to reveal how the introgressed regions of the genome have adapted to the host environment.

### Autosomal data support a monophyletic origin of *N. tetrasperma*

Based on the observation of unrelated histories of the *mat* chromosomes in certain lineages of *N. tetrasperma*, Metzenberg and Randall (1995) hypothesized that *N. tetrasperma* is an ephemeral species that has arisen repeatedly by the associations of heterothallic strains of different mating type [83]. In contrast, in the study by Menkis et al. (2009), phylogenetic analyses of a concatenated dataset consisting of four unlinked microsatellite flanking regions indicate a monophyletic origin of *N. tetrasperma* [38]. However, in the study by Menkis et al. (2009), as in this study (Figure 4, Figure S3), discordance between the gene trees reconstructed from single autosomal loci was found, which could be the result of various biological processes including introgression, hybridization and incomplete lineage sorting (ILS). Our results from Bayesian concordance analysis (BCA) for the autosomal data show support for the monophyly of *N. tetrasperma*, but are consistent with rapid speciation in the heterothallic Neurospora clade, and suggest ILS as the cause of the observed discordance. Specifically, in our analysis, the concordance factors for all the clades were low, i.e., less than 0.5 for all clades (excluding *N. tetrasperma* homokaryotic stains from the same heterokaryon) (Table S7), indicating that there exist a low level of concordance for the loci and taxa in this study. This result contrasts with the BCA by Ané et al. (2007) on eight species of *Saccharomyces*, where the concordance observed for 106 nuclear genes was very high [69], [84]. The relatively low level of concordance among loci in this study may be due to the rapid speciation within the heterothallic clade of Neurospora, or to the action of introgression between species. Indeed, it has previously been noted that the time between speciation events was likely compressed in the heterothallic clade of Neurospora (i.e., a short time between speciation events) [40]. Nevertheless, a clear pattern for the three clades presented for the autosomes show the *N. tetrasperma* clade as the dominant history (Figure 5, Figure S6), supporting a monophyletic history of *N. tetrasperma*.

### *mat a*–specific introgression and structural heterozygosity of the *mat A* chromosome

One of the most remarkable results presented herein is the bias of introgression: it was only detected in the *mat a*, and not *mat A*, chromosomes for each of the investigated lineages of *N. tetrasperma*. The biased introgression in *N. tetrasperma* might be attributable to the genomic architecture of the *mat* chromosomes in this taxon. Specifically, by performing reciprocal introgression of *mat* chromosomes between *N. tetrasperma* and *N. crassa*, Jacobson (2005) found that the *mat a* chromosome of the investigated strain of *N. tetrasperma* (P581, L6) is collinear with the *N. crassa mat* chromosomes, whereas the *mat A* chromosome is structurally different [44]. This finding lead him to propose that structural rearrangements on the *mat A* chromosome had occurred in *N. tetrasperma* and were correlated with, and possibly the cause of, suppressed recombination between the *mat* chromosomes in this taxon [44]. His findings were further supported by a subsequent study by Ellison et al. (2011), in which the authors used high quality genome assemblies of the single mating-type component strains of strain P581 (L6) to discover a series of three inversions on the *mat A* chromosome [49]. It is not possible to reveal rearrangements in the *mat* chromosome with the data presented in this study. However, assuming that rearrangements of the *mat A* chromosome is inherent to, and the cause of suppressed recombination, in all strains of *N. tetrasperma*, one may speculate that the introgression into *N. tetrasperma* revealed in this study is the result of hybridization events between a homokaryotic self-sterile *mat A* strain of *N. tetrasperma* and a *mat a* strain of a closely related heterothallic species, followed by subsequent backcrossing into the *N. tetrasperma* population. The structural heterozygosity between the *mat* chromosomes of the parents of such hybridization would result in the preservation of the introgression tract in the *mat a* chromosome during the backcrossing with the *N. tetrasperma* population, while the signal would be lost over time in the pseudoautosomal flanks and autosomes due to the action of recombination. One may also speculate that introgression, rather than the rearrangements, are the cause of suppressed recombination in the strains included in this study. However, the finding that the region of divergence is larger than the region of introgression, especially in L1 (Figure 3) does not fit well with this hypothesis.

### Possible scenarios explaining the evolutionary history of the *mat* chromosomes in *N. tetrasperma*

**Figure 8:**
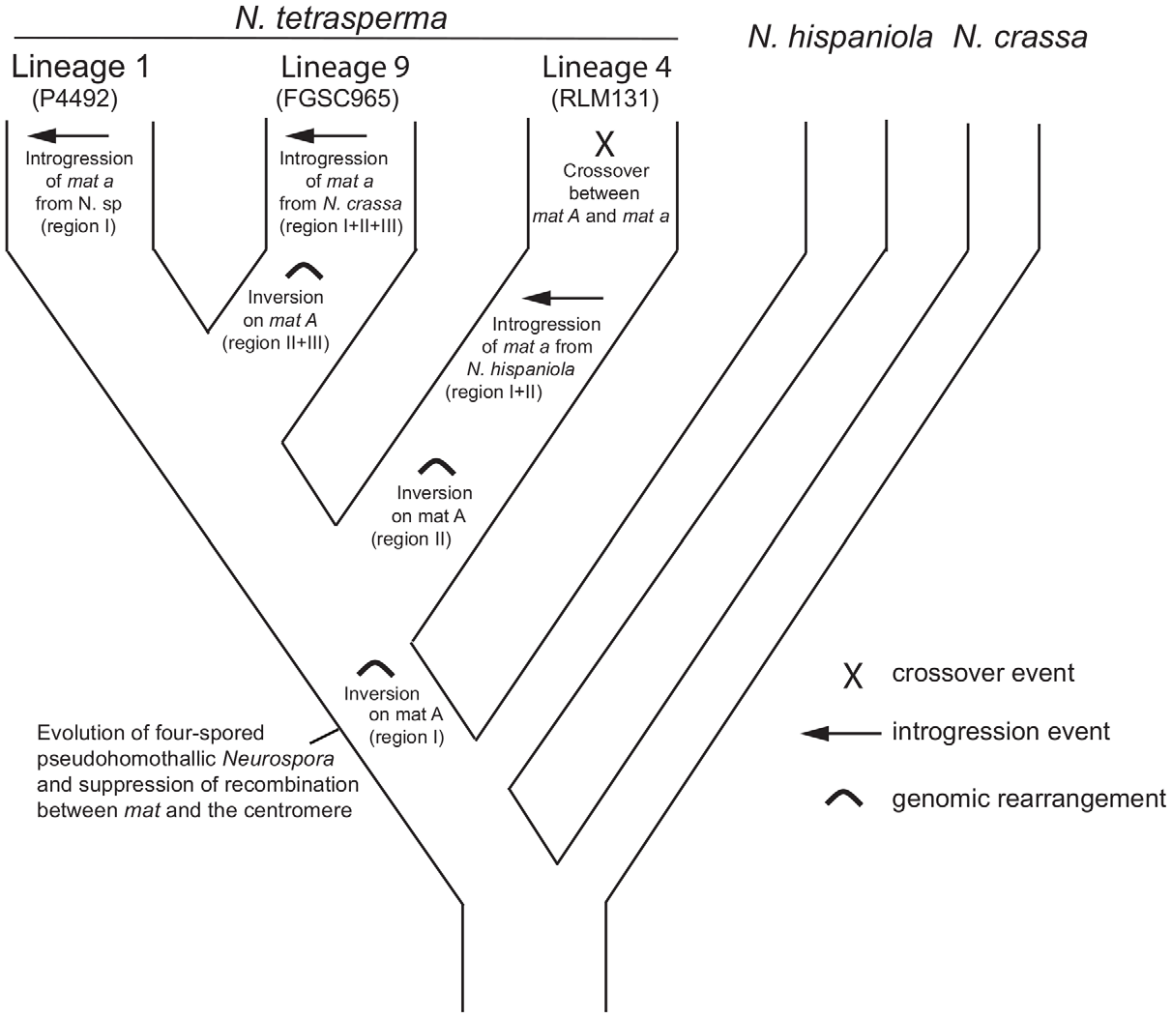
A possible scenario explaining the evolution of the mating-type (*mat*) chromosomes in lineages of *N. tetrasperma*. A putative scenario for the evolutionary processes leading to the results observed in this study. We speculate that a rearrangement on the *mat A* chromosome was associated with the evolution of pseudohomothallism in *N. tetrasperma*. During the subsequent divergence of the *N. tetrasperma* lineages, additional rearrangements, introgressions and a crossover took place.

By using divergence data between alleles of the two *mat* chromosomes in *N. tetrasperma* strain P581 (L6), Menkis et al. (2008) suggested that recombination cessation on the *mat* chromosomes of *N. tetrasperma* has taken place in two or more successive events, so called “evolutionary strata” [35]. Divergence estimates suggest that the first event was contemporaneous with the split of *N. tetrasperma* from a common ancestor with *N. crassa*, while the second occurred more recently [35]. In line with this finding, a more recent study by Menkis et al. (2010) revealed an independent expansion of the recombination block in the different, reproductively isolated, lineages of *N. tetrasperma* [63]. The recent publication by Ellison et al. (2011) supports the notion that the region of suppressed recombination of the *N. tetrasperma mat* chromosomes has grown over evolutionary time [49]. They suggest that the first evolutionary stratum identified by Menkis et al. (2008) corresponds to two large inversions on the *mat A* chromosome of strain P581(L6), and that at least one smaller inversion have taken place more recently. Based on these previous reports, we propose a scenario for the events leading to the results observed in this study. This scenario is schematically depicted in Figure 8. First, we propose that the evolution of pseudohomothallism in *N. tetrasperma* was associated with an inversion of the *mat A* chromosome. This first inversion corresponds to the region I shown Figure 3 and also to one of the two earliest inversions found by Ellison et al. 2011 [33] to have occurred on the *mat A* chromosome of the *N. tetrasperma* strain P581 (L6). After this, the lineage containing strain RLM131 (L4) diverged, as indicated by both the primary concordance tree and the phylogeny of autosomal genes (Figure 1, Figure S6), where after a lineage specific second inversion occurred on the *mat A* chromosome. This second inversion corresponds to region II in Figure 3, and to the second early inversion found by Ellison et al. (2011) [33] to have occurred in strain P581 (L6). Subsequently, a *mat A* homokaryotic strain of L4 hybridized with a *mat a* strain of *N. hispaniola*, and since these two strains exhibit structural heterozygosity on the *mat* chromosomes, the signal of introgression from *N. hispaniola* was preserved in the *mat a* chromosome during the subsequent backcrossing into the *N. tetrasperma* population. After the introgression, a crossover event between the two *mat* chromosomes (previously reported by Menkis et al. 2010 [63]) lead to the transfer of introgressed DNA from the *mat a* to the *mat A* chromosome, and gave rise to the result found in the BCA analyses (Figure 7). After the split of lineage 9 from lineage 1, additional inversion(s) occurred in this lineage, corresponding to region II and III in Figure 3, whereafter the region of the *mat a* chromosome corresponding to region I, II and III in Figure 3, is introgressed from *N. crassa*. In lineage 1 no additional inversions are found and the region I is introgressed from a heterothallic taxon not specifically identified in this study. This scenario fits with previous reports on the growth of the region of suppressed recombination of the *mat* chromosomes of *N. tetrasperma* [35], [63], and the diversification of reproductively isolated lineages of *N. tetrasperma*, over evolutionary time [38]. However, our assumption of an early inversion on the *mat A* chromosome, corresponding to region I in Figure 3, occurring before the divergence of the *N. tetrasperma* lineages, contradicts the model of order of inversions found in strain P581 (L6) proposed by of Ellison et al. 2011 [49]; although the two first inversions were not distinguishable in time, they propose that the inversion corresponding to region I was the second inversion occurring in this strain. Further studies on *mat* chromosome synteny of multiple *N. tetrasperma* lineages, which requires higher coverage genomic data, are needed to further shed light on the cause and order of events leading to the observed results.

### Conclusion

In the present study, we reported large-scale *mat a* specific introgression from heterothallic Neurospora species to the pseudohomothallic *N. tetrasperma*. The introgression tracts range from 4.1 to 5.6 Mbp in size, and are located within the divergent region of the *mat* chromosome for all three *N. tetrasperma* lineages, while no signal of introgression is found in the rest of the genome. Molecular evolution analyses indicate that the introgression from freely recombining species of Neurospora is associated with a lower level of degeneration for the *mat a* chromosome. Future analysis of population data from multiple lineages of *N. tetrasperma* will be required to estimate how frequent and widespread introgression has been in shaping the genome evolution of *N. tetrasperma* and its close relatives. Taken together, the results presented here can be used to infer various features of the genomic evolution in *N. tetrasperma*, and its novel mating system. Furthermore, we argue that the results presented here, using an alternative fungal model system, contribute towards a greater general understanding of the evolutionary significance of introgression in natural populations of higher eukaryotes.

## Materials and Methods

### Fungal material

All strains of Neurospora used herein are listed in Table S1. The strains were obtained from the Fungal Genetics Stock Center (FGSC), University of Missouri, Kansas City, USA, with the exception of the haploid, single mating-type component strains of heterokaryon 965, which were isolated from asexual spores produced by the heterokaryon in our laboratory [38].

### Growth conditions and DNA extraction

Strains for DNA extraction were grown in 18×200 mm culture tubes containing minimal medium broth (Vogel, 1964) with 1% sucrose. We extracted DNA from fungal vegetative tissue using a CTAB-based extraction protocol previously described [85], or by the Easy-DNA Kit (Invitrogen, Carlsbad, CA).

### Genome sequencing

Each of the six haploid genomes was sequenced by Illumina's Genome Analyzer II platform at Geneservice, Source BioScience plc group (http://www.geneservice.co.uk/home/). The paired-end DNA library construction and sequencing process followed the Illumina's Genome Analyzer II Operation Guide. The Illumina reads are the standard 2×55 bp paired-end reads with insert size of ∼170 bp. Illumina quality scores were sampled (1 out of 1000 reads) and calculated to get the read quality for each genome. The average quality of each base position was calculated based on Illumina Fastq data, and the error rate was estimated based on the quality score. Reads are available at the Sequence Reads Archive at NCBI (http://www.ncbi.nlm.nih.gov/sra/) under the accession numbers SRR099270 (L1A), SRR099222 (L1a), SSR363378 (L4A), SSR363379 (L4a), SSR363376 (L9A) and SSR363377 (L9a). The reads from two of the genomes (L1A and L1a) were used in a previous study to generate gene sequence data for 207 genes [47].

### Genome assembly

All Illumina reads from each *N. tetrasperma* genome were mapped against the reference genome of *Neurospora crassa* (finished release version10, sequenced by Broad Institute, http://www.broadinstitute.org/annotation/genome/neurospora/Home.html) by the assembly program MOSAIK (version 1.01370: http://bioinformatics.bc.edu/marthlab/Mosaik). *Neurospora crassa* was chosen as a reference genome because of its close phylogenetic relationship to *N. tetrasperma* (∼4% nucleotide difference), high quality of genome sequence (20 supercontigs, <1% gaps), structural collinearity with the *N. tetrasperma mat a* genome and representative of a structural ancestral stage. The maximum allowed number of mismatches for each read was set to 10 (i.e., 18%). The seeding hash size was set to 12, alignment candidate threshold to 20 bp and hash position threshold set to 100, to obtain a high quality of assembly. For all the other settings we used the program defaults. The assembly output files were converted from the assembly storage format SAM [86] to BAM (http://iesdp.gibberlings3.net/file_formats/ie_formats/bam_v1.htm), whereafter the BAM files were sorted and indexed according to the genomic coordinate by using the program Samtools (version 0.1.7: [86]). Consensus bases were called from the sorted BAM format assembly file by Samtools, using minimum reads depth of 3 and min RMS mapQ of 25. Consensus sequences were constructed from the consensus bases called from each of the six genome-assemblies, and their genomic coordinate was strictly following the reference genome. Regions of the reference genome not covered with reads were treated as missing data in the consensus sequence. The coverage for each genome was calculated by MOSAIK.

### Sequencing quality and assembly coverage

For each of the genome, the error rate was lower than 0.3%, calculated based on average Illumina quality score (Table S2). In addition, we manually blasted the DNA sequence of two nuclear genes located on the *mat* chromosome (*lys-3* and *upr-1*), which were acquired from each of the genome by PCR sequencing in a previous study [35], against each of our consensus sequences. Only 1 bp mismatch was found out of 72,360 bp checked in total, thus the estimated error rate is 0.0014% on the consensus level in these two loci. The coverage for the six sequenced genomes ranges from 12.0X to 19.2X (Table S7). For each genome, the whole genome coverage is slightly higher than the *mat* chromosome coverage (Table S2). This could be caused by a sequencing bias, or that in the assembly step, reads were more difficult to map to *mat* chromosome of *N. crassa* reference compared to autosomes, since the *mat* chromosome constitutes a hotspot for nucleotide and structural variation [33].

### Extraction of gene sequences from consensus genomes of *N. tetrasperma*

Nuclear genes were acquired by blasting *N. crassa* transcripts data (http://www.broadinstitute.org/annotation/genome/neurospora/MultiDownloads.html) against the consensus sequences of the six *N. tetrasperma* lineages. In total, 2,521 genes with complete CDS in all six genomes were extracted from the blast output and selected for further analyses; 1,978 autosomal genes and 543 from the region of the *mat* chromosomes subjected to introgression in all strains of *N. tetrasperma* studied here.

### Alignment and divergence estimation

A seven-way genomic alignment was constructed from the reference genome (*N. crassa*) and the six consensus genomes acquired in this study (*N. tetrasperma* L1A, L1a, L4A, L4a, L9A, L9a). At the stage of data analysis, the genome of the *N. tetrasperma* strain P581 (belonging to lineage L9 [38]) at the joint genome institute (http://www.jgi.doe.gov/) was still embargoed, and was therefore not included in the analyses. All the columns with at least one gap in the alignment were removed by trimAl (version 1.2), a tool for automated alignment trimming. After the removal of gapped-columns, the alignment length of the *mat* chromosome (LGI in *N. crassa*) was reduced from 9,798,893 bp to 7,657,888 bp, and the length of the trimmed alignments from the additional six chromosomes (referred to in this study as autosomes) were 3,236,265 bp (4,478,683 bp before the removal of gaps) for LGII, 4,010,562 bp (5,274,802 bp) for LGIII, 4,395,417 bp (6,000,761 bp) for LGIV, 5,009,410 bp (6,436,246 bp) for LGV, 3,049,455 bp (4,218,384 bp) for LGVI and 2,993,263 bp (4,255,303 bp) LGVII. The pair-wise divergences between the genomes were calculated as the fraction of differences in bp between the sequences, using either a sliding window over the seven-way alignment or a comparison of the complete consensus sequences for a chromosome. As the observed pair-wise differences between *N. crassa* and *N. tetrasperma* is low, for most regions below 5%, corrections for multiple hits were not required [87]. Window size was set to either 500 or 100 kb, with step sizes of 100 or 20 kb, respectively, based on the purpose of the analysis.

### GC content and gene density

GC content was calculated with a window size set to 100 kb and step size of 20 kb for *N. crassa* and *N. tetrasperma* L1A. Gene position in L1A was plotted based on the hit position of blasting all *N. crassa* genes (*Neurospora crassa* release 10, http://www.broadinstitute.org/annotation/genome/neurospora/MultiDownloads.html) against consensus genome of L1A.

### Demarcations of regions of elevated sequence divergence and introgressions along the *mat* chromosomes

A step-wise approach was used in order to pinpoint the borders between the region of elevated divergence and the flanking pseudoautosomal (PA) regions of the *mat* chromosomes. First, the pair-wise alignments were divided into non-overlapping sections with length of 100 kb, and the percent nucleotide differences for each section was calculated. Based on the level of divergence observed for the autosomes (mean ∼0.46%, peak<1%), a cutoff was set to 1% nucleotide difference to separate the PA regions from the region of elevated divergence of the *mat* chromosome. Second, the alignment sections (100 kb) near the border of PA regions were further divided into smaller sections (10 kb), in the purpose of more specifically pin-point the borders using the same cutoff of 1%. The flanking regions of the *mat* chromosome with average nucleotide difference lower than 1% was assigned as PA1 and PA2 region, respectively, and the central part as the region of elevated sequence divergence.

Regions likely to be subjected to introgression were determined by the same step-wise approach (100 kb, 10 kb) in pair-wise comparisons of each putative introgressed strain and a non-introgressed reference strain (965A or *N. crassa*).

### Statistical analyses of sequence divergence

A binomial sign test was used to statistically test for a correlation between the divergences of the pair-wise samples along the *mat* chromosome. This is an exact test of the statistical significance of deviations from a theoretically expected distribution. The null hypothesis is that all pair-wise divergences will be correlated along the chromosomes, unless introgression has brought in genetic material into certain regions that distort the divergence correlation.

### Phylogenetic analyses of loci along the *mat* chromosomes and autosomes

A maximum likelihood phylogenetic analysis was carried out with RaxML 7.0.1 [88] on the concatenated dataset of 1,978 autosomal genes and the 543 genes from the *mat* chromosome, from the six *N. tetrasperma* genomes (L1A, L1a, L4A, L4a, L9A and L9a) and *N. crassa*. The best fitting nucleotide substitution model was GTRGAMMAI, inferred by jModeltest 0.1.1 [89]. Bootstrap replicates run 100 times to give the support value.

Single locus phylogenies were generated from 10 autosomal loci and 16 loci on the *mat* chromosome (Figure 3, Table S6), selected as evenly distributed across the genome/chromosomes. For eight selected loci, in addition to the six *N. tetrasperma* genomes, we included representatives of all known heterothallic taxa of Neurospora (Table S1) in the analysis. Four of these gene loci are located on the *mat* chromosome of Neurospora (*nit-2*, *eth-1*, *cys-9* and *phr:* marked in bold in Figure 3) and of these, two loci (*eth-1* and *cys-9*) are found inside the region of introgression for all three *N. tetrasperma* strains, and two loci (*nit-2* and *phr*) in the left and right PA regions, respectively (Figure 3). For the additional loci of Table S6, we included a subset of the known heterothallic taxa of Neurospora (Table S6). For all 16 analyzed loci on the *mat* chromosomes (Table S6), data from heterothallic strains were generated specifically for this study, alignments are available from TreeBASE (http://purl.org/phylo/treebase/phylows/study/TB2:S12093), while for the autosomal loci we used available data (Table S6). Description of the loci, specific primers for their amplification, PCR conditions and procedures for DNA sequencing can be found in Menkis et al. (2008) [35], with the exception of *mat A-1* and *mat a-1*, which were amplified with the primers matA F1+R4 and mat aF1+R9, respectively, as described in Wik et al. (2008) [91]. The generated sequences were edited with Sequencher 4.7 (Gene Codes Corporation, Ann Arbor, MI) and aligned using MUSCLE V3.6 [90] with the default settings. The additional four loci for which representatives of all heterothallic species were included are microsatellite-flanking loci (DMG, QMA, TMI and TML) located on different autosomal chromosome arms: The DMG locus is on linkage group (LG) IVR, QMA is on LG IIR, TMI is on LG VR and TML is on LG VL. We generated sequence alignments for these autosomal loci by obtaining sequence data from previously published datasets [38], [40], [92]. In addition, we performed phylogenetic analyses on additional gene loci, for which data were available for a subset of heterothallic species (Table S1, Table S6).

To reconstruct the phylogenetic histories of each of the loci, we performed both Maximum likelihood (ML) and Bayesian phylogenetic analyses. The statistical selection of the best-fit nucleotide substitution model was completed using the Akaike Information criterion, as implemented in jModeltest 0.1.1 [89]. The ML phylogenetic analysis was carried out for each locus using Garli v1.0 [93]. Each of the ML phylogenetic analyses was run with five independent replicates to ensure that the search was not trapped in a local optimum. Support for the nodes of the ML tree was estimated using non-parametric bootstrap analysis implemented in Garli v1.0. We carried out 500 bootstrap replicates for each gene tree analysis. Bayesian phylogenetic analysis of each locus was carried out using MrBayes v3.1.2 [94], [95]. Two independent runs with 4 chains in each were run in MrBayes for more than 35 million generations each, with a sample frequency of every 1000 generations. To test for convergence, we examined trace plots of the likelihood scores for trees sampled by the Markov chain using Tracer v1.5 [96]. We also used the compare function in AWTY [97] to graphically test for convergence between the two independent runs. The first 25% of generations were discarded as burnin for each analysis.

### Bayesian concordance analysis

Bayesian concordance analysis [65] was carried out using 16 loci from across the *mat* chromosomes and 10 loci from the autosomes, for the six *N. tetrasperma* genomes and representatives of 4 heterothallic species (Table S6). Bayesian phylogenetic analysis for each of the 26 loci was carried with MrBayes v3.1.2 [94], [95]. All analyses were run for no less than 5 million generations with a sample frequency of every 1000 generations. Convergence was determined by ensuring that the average standard deviation of split frequencies was less than 0.002 for each of the individual analysis. Bayesian concordance analysis was carried out in BUCKy v1.4.0 [64] using the posterior distributions of trees generated using MrBayes as described above. Each concordance analysis was run with different priors on alpha (0.001, 0.1, 1.0, 10, 100, ∞) to determine the effect of the prior on the analysis. Each BUCKy analysis was carried out separately for the autosomal loci and loci on the two *mat* chromosomes (*mat A* and *mat a*). The concordance analyses were run with 2 independent runs of four chains each. The chains were 10,000,000 generations in length with the first 10% of the chain discarded as a burn-in. Convergence of the runs was determined by ensuring that the standard deviation of concordance factors was less than 0.002.

### Analyses of the ratio of nonsynonymous to synonymous substitutions (dN/dS) among branches

We used the maximum likelihood based program codeml included in the PAML (Phylogenetic Analysis by Maximum Likelihood) package, Version 4.3, to test different hypotheses of the ratio of non-synonymous to synonymous substitution rates (dN/dS, or ω) in the introgressed region of *N. tetrasperma*. For this test, we used the concatenated data from the 543 genes mentioned above. The input phylogenetic tree for codeml was (L4a,(L4A,L1A,L9A),(L9a,crassa),L1a), which was the phylogeny obtained by analyzing the 543 concatenated genes by RaxML (see above). We implemented the global ω (one-ratio) model in codeml, assuming the same ω for all branches in the phylogeny, the free-ratio model in which each branch in the phylogeny is allowed an independent ω, and local models of ω to test for alternative hypotheses of unequal ω rates in *mat A* and *mat a* chromosomes of *N. tetrasperma*. The relative fit of the data to different nested models were tested by using likelihood-ratio test where twice the difference in log likelihood values (-2lnΔ) was compared using a χ^2^ distribution with one degree of freedom.

First, we tested for an unequal ω between the two *mat* chromosomes of the same heterokaryon. For example, for strain RLM131 we investigated the relative fit of our data to the two nested local models (L4a#1,(L4A#1,L1A,L9A),(L9a,crassa),L1a) and (L4a#1,(L4A#2,L1A,L9A),(L9a,crassa),L1a). Second, we tested for a general difference of ω between *mat A* and *mat a* chromosomes of *N. tetrasperma*, by investigating the relative fit for the data to the global model as compared to a local model in which the branches delineating *mat A* chromosomes were allowed to be different from the branches delineating *mat a* chromosomes, i.e., (L4a#1,(L4A#1,L1A#1,L9A#1)#1,(L9a#1,crassa)#1,L1a#1). vs. (L4a#2,(L4A#1,L1A#1,L9A#1)#1,(L9a#2,crassa)#2,L1a#2).

### Analyses of codon usage bias for genes in the introgressed region

We obtained the preferred codon usage table for *N. crassa* from Codon Usage Database (http://www.kazusa.or.jp/codon/), and further confirmed by RSCU values that the *N. tetrasperma* use the same 21 preferred codons as in *N. crassa* (Table S9). The concatenated 543 complete genes were used to calculate allele specific switches from preferred (PR) to non-preferred (NPR), non-preferred (NPR) to preferred (PR), and frequency of excess PR to NPR switches per lineage.

## Supporting Information

Figure S1 Sliding window coverage depth for seven chromosomes of all six haploid genomes of *Neurospora tetrasperma* in this study. The thicker lines represent 400 kb window size (step size 400 kb) sliding along the chromosome; thinner lines 100 kb (100 kb).
(PDF)

Figure S2 Pair-wise divergences and GC estimates of the six autosomes (LGII-LGVII) of *Neurospora tetrasperma* and *Neurospora crassa*. A: Intra-lineage comparisons of *N. tetrasperma*. The lines represent 500 kb window size (step size 100 kb) sliding along the chromosome, B: Comparisons of genomes from different lineages/species. Solid lines represent *N. tetrasperma*-*N. crassa* comparisons and dashed lines represent *N. tetrasperma-N. tetrasperma* comparisons. All lines represent 500 kb window size (step size 100 kb) sliding along the chromosome. C: GC content in *N. crassa* (black line) and *N. tetrasperma* L1A (red line). Window size is 100 kb, step size 20 kb. Black bars indicate gene density in L1A.
(PDF)

Figure S3 Gene genealogies for six autosomal gene loci of Neurospora. Each tree includes three *N. tetrasperma* lineages (red), and four heterothallic Neurospora species (blue). *Neurospora discreta* was used as the outgroup in all analyses. The topologies shown are from the Bayesian phylogenetic reconstruction with the posterior probabilities (as a percentage) from the analysis shown above the branches.
(PDF)

Figure S4 Gene genealogies for 13 genes on the mating-type (*mat*) chromosome of Neurospora. Each tree includes three *N. tetrasperma* lineages (red), and four heterothallic Neurospora species (blue). Strain IDs are shown in Table S6. *Neurospora discreta* was used as the outgroup in all analyses. The topologies shown are from the Bayesian phylogenetic reconstruction with the posterior probabilities (as a percentage) from analysis shown above the branches.
(PDF)

Figure S5 The Concordance Factors of the Bayesian Concordance Analysis for 6 different choices of prior discordance. The alpha priors are from left to right as follows: 0.001, 0.1, 1.0, 10, 100 and ∞.
(PDF)

Figure S6 Primary concordance tree for autosomal genes of Neurospora strains included in the BCA. The numbers displayed above the branches represent the concordance factors with the 95% credibility interval in parenthesis.
(PDF)

Table S1 Strains of Neurospora used in the study.
(PDF)

Table S2 Assembly statistics for six haploid genomes of *Neurospora tetrasperma*.
(PDF)

Table S3 Pair-wise nucleotide differences, estimated as the fraction of different nucleotides, for the *mat* chromosome and autosomes of six haploid genomes of *Neurospora tetrasperma*, and *Neurospora crassa*.
(PDF)

Table S4 Boundaries and nucleotide divergences for regions of the mating-type (*mat*) chromosomes originating from three *Neurospora tetrasperma* wild-type heterokaryons.
(PDF)

Table S5 Pair-wise nucleotide differences, estimated as the fraction of different nucleotides, of different regions of the mating-type (*mat*) chromosome (LGI). Data from pseudoautosomal (PA) regions are given as the PA regions shared between all heterokaryons of *N. tetrasperma* PA1: (position 1–900,000), the central region introgressed in all three heterokaryons (1,480,000–5,450,000) and PA2 shared between the heterokaryons (7,000,000–7,657,888).
(PDF)

Table S6 Information on loci and heterothallic strains of Neurospora used in gene tree and Bayesian Concordance analyses.
(PDF)

Table S7 Concordance factor (CF) and 95% credibility interval estimated for clades in BCA analysis of autosomal loci. The table shows only clades with concordance factor greater than 0.05.
(PDF)

Table S8 Concordance factor (CF) and 95% credibility interval estimated for clades in BCA analysis of loci on the mating-type (*mat*) chromosomes. The table shows only clades with concordance factors greater than 0.05.
(PDF)

Table S9 The preferred codons for *Neurospora tetrasperma* and *Neurospora crassa*.
(PDF)
