# Supplementary figures and images for: Large-Scale Introgression Shapes the Evolution of the Mating-Type Chromosomes of the Filamentous Ascomycete Neurospora tetrasperma
Source: PLoS Genet. 2012 Jul 26;8(7):e1002820. doi: 10.1371/journal.pgen.1002820 (PMC3406010; doi:10.1371/journal.pgen.1002820)

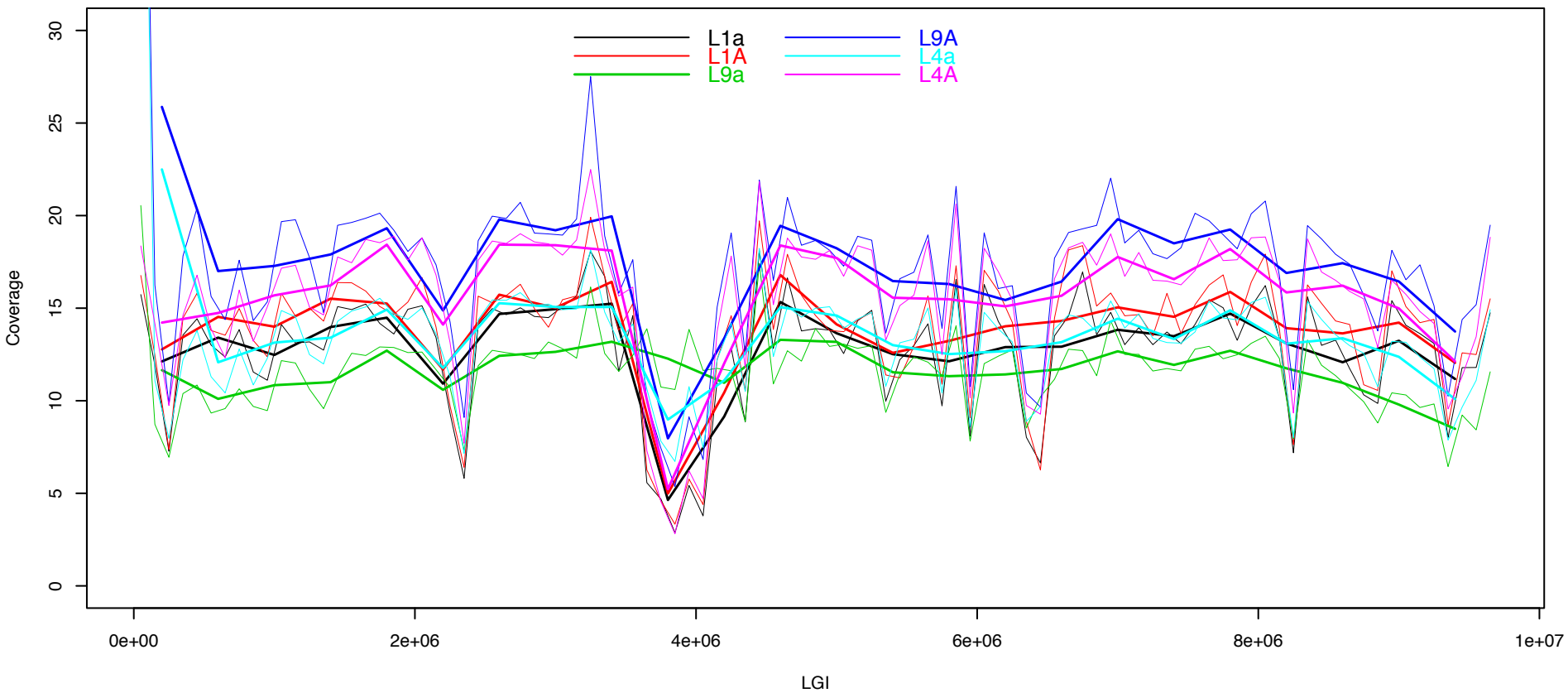

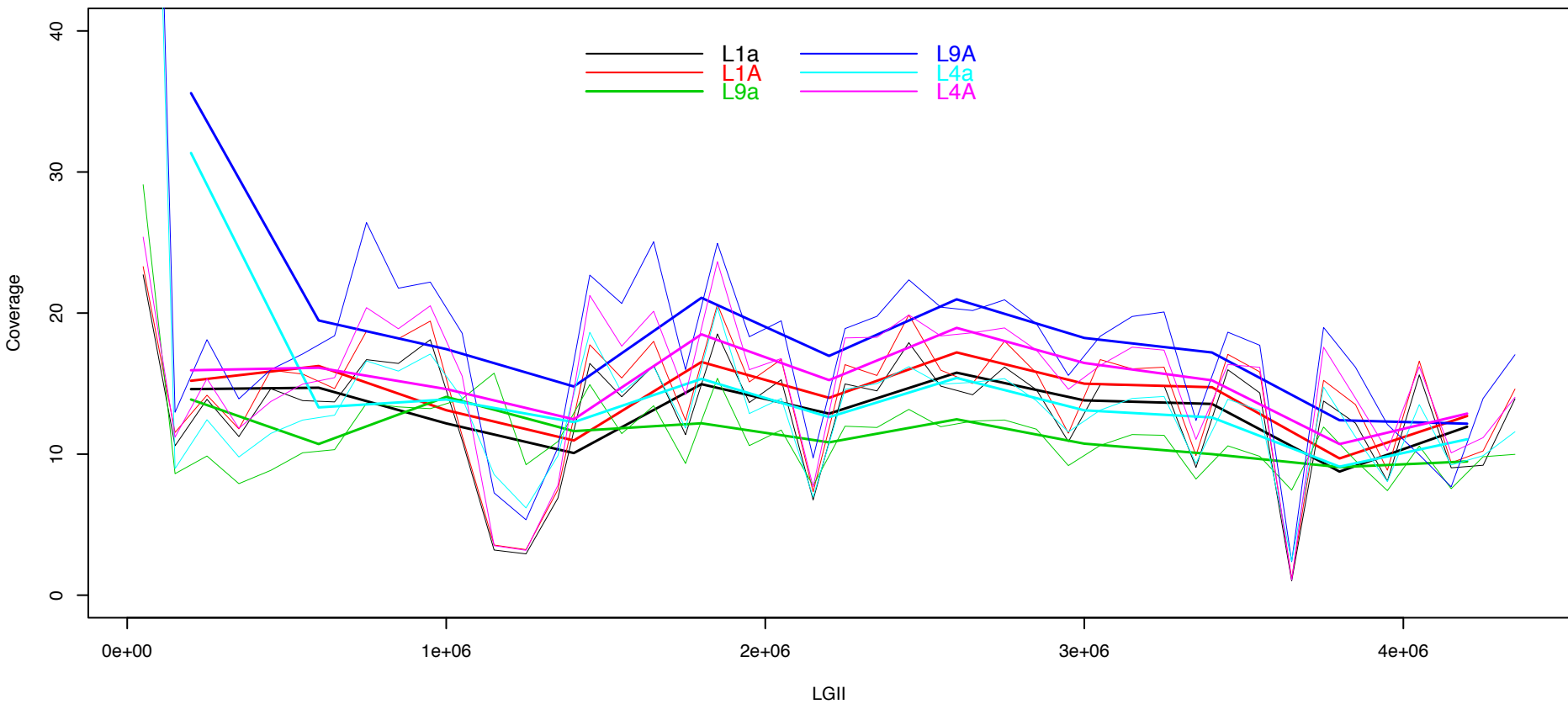

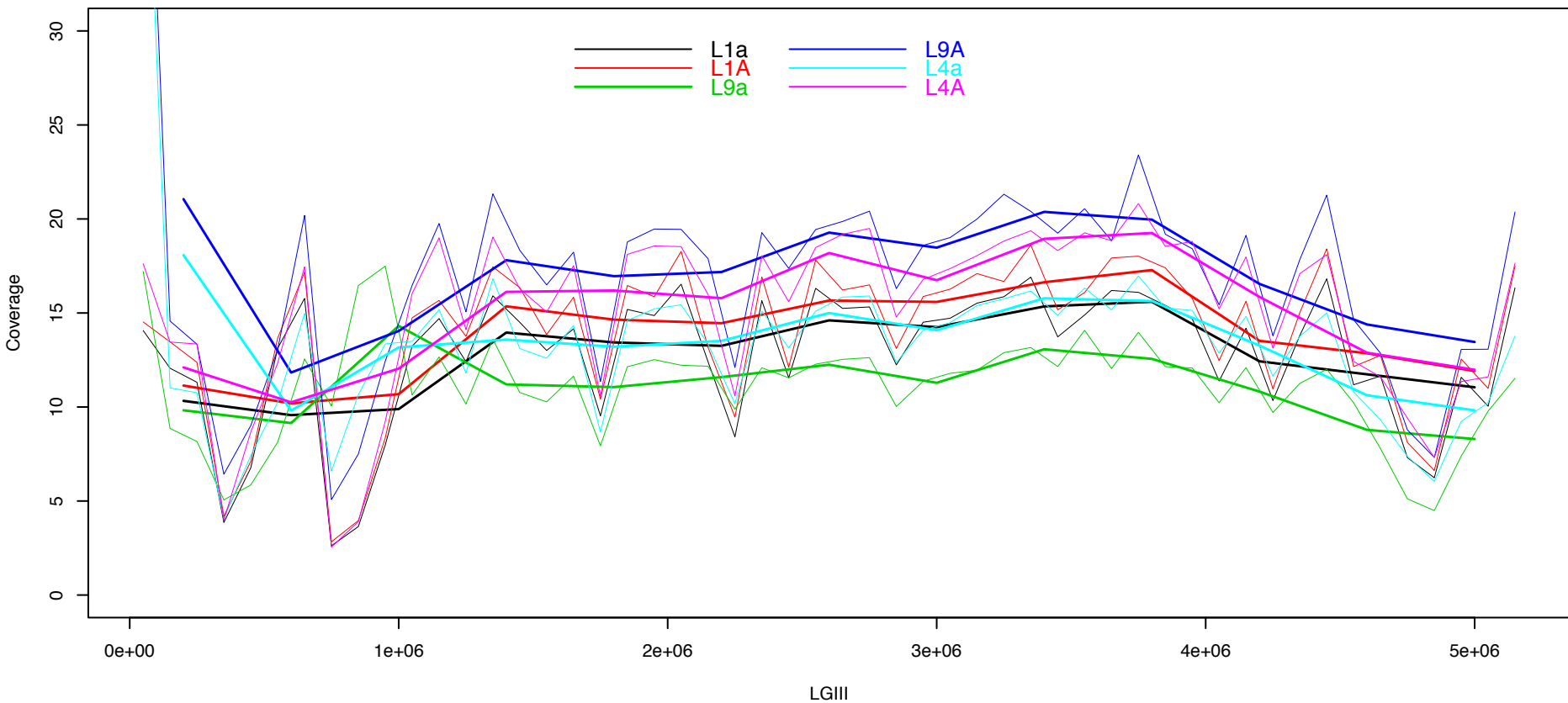

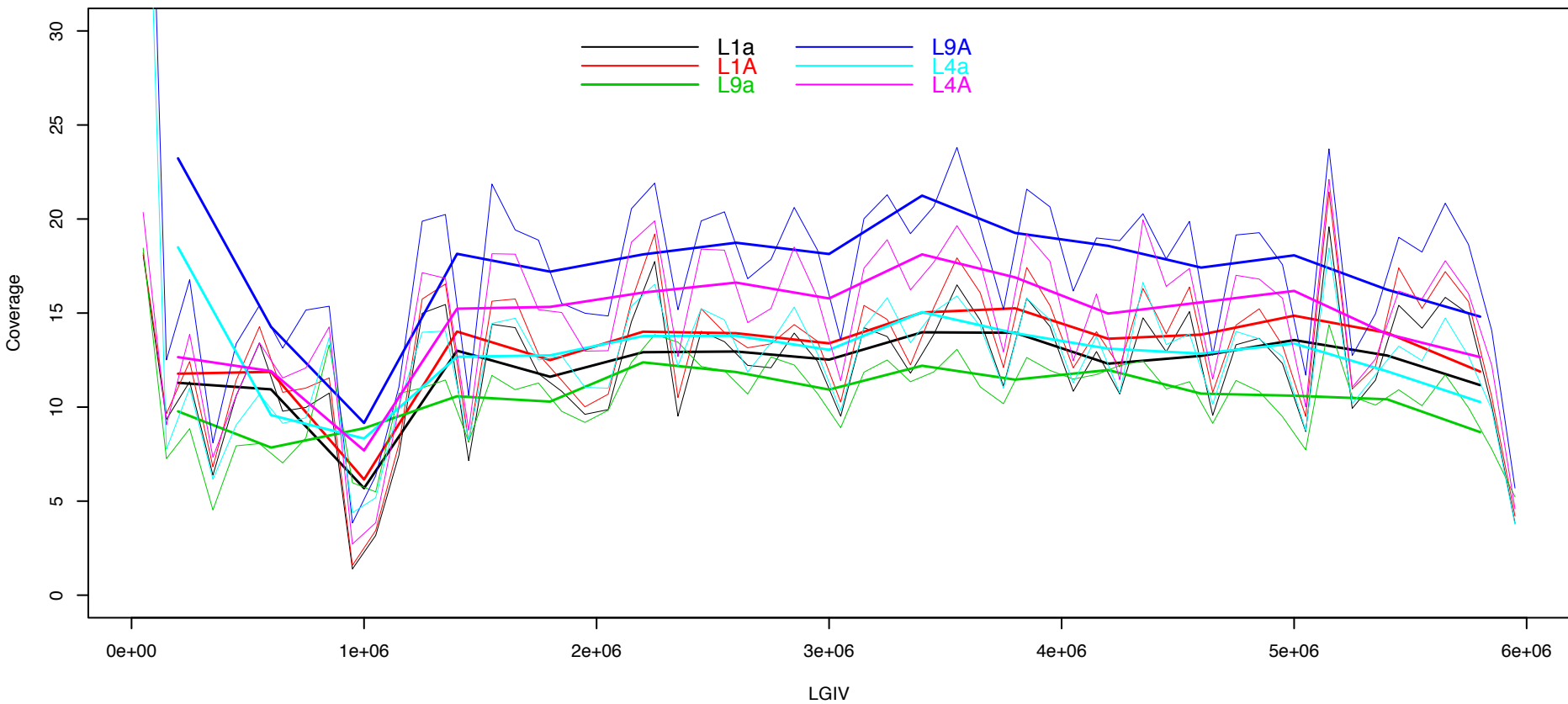

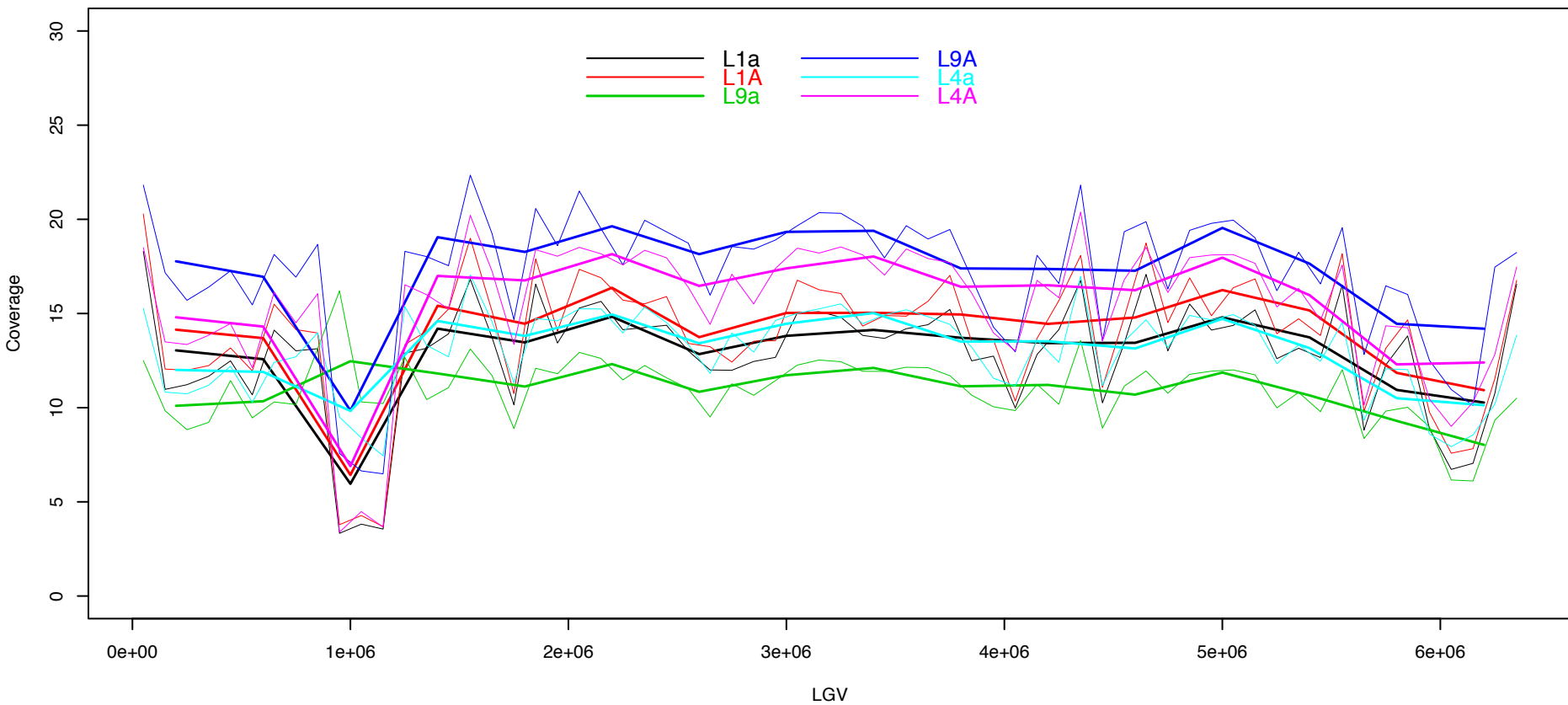

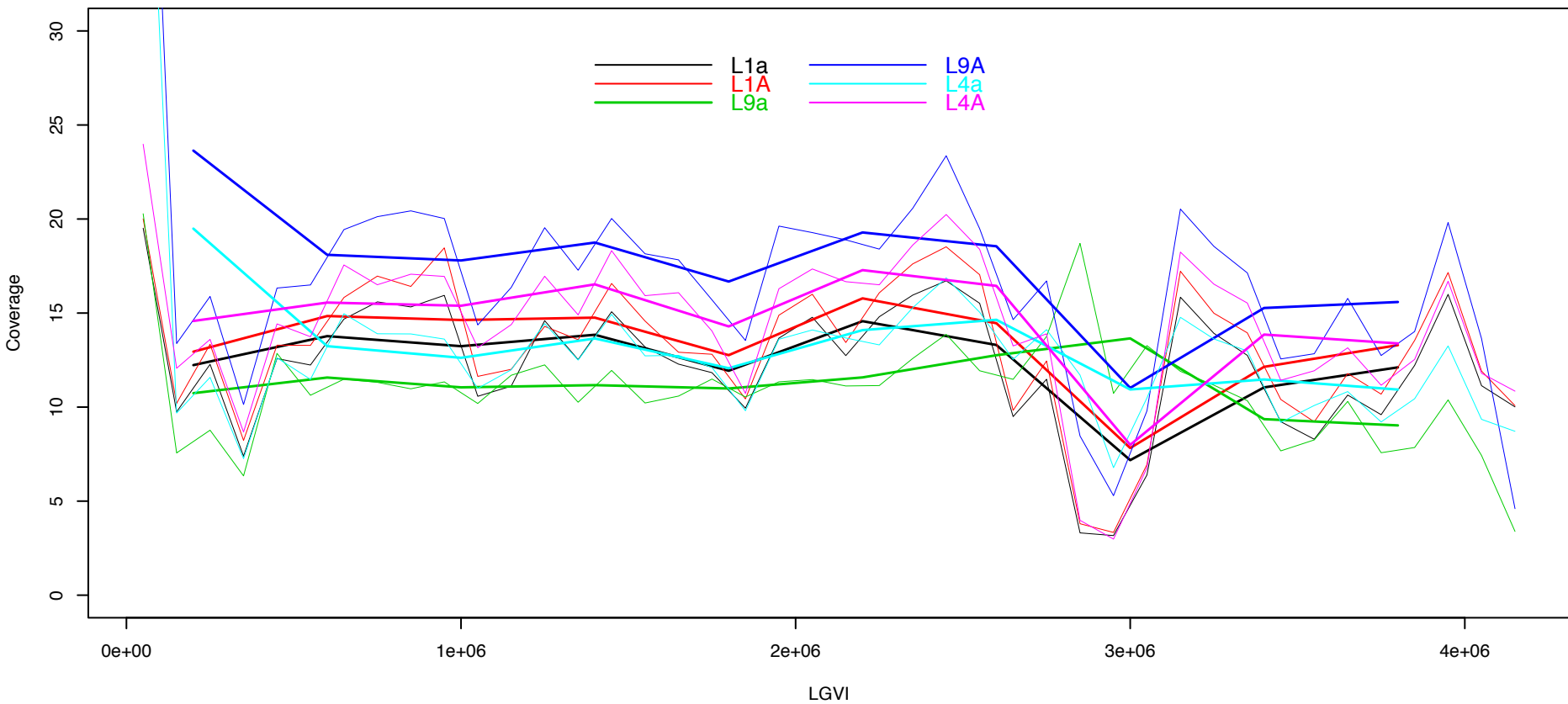

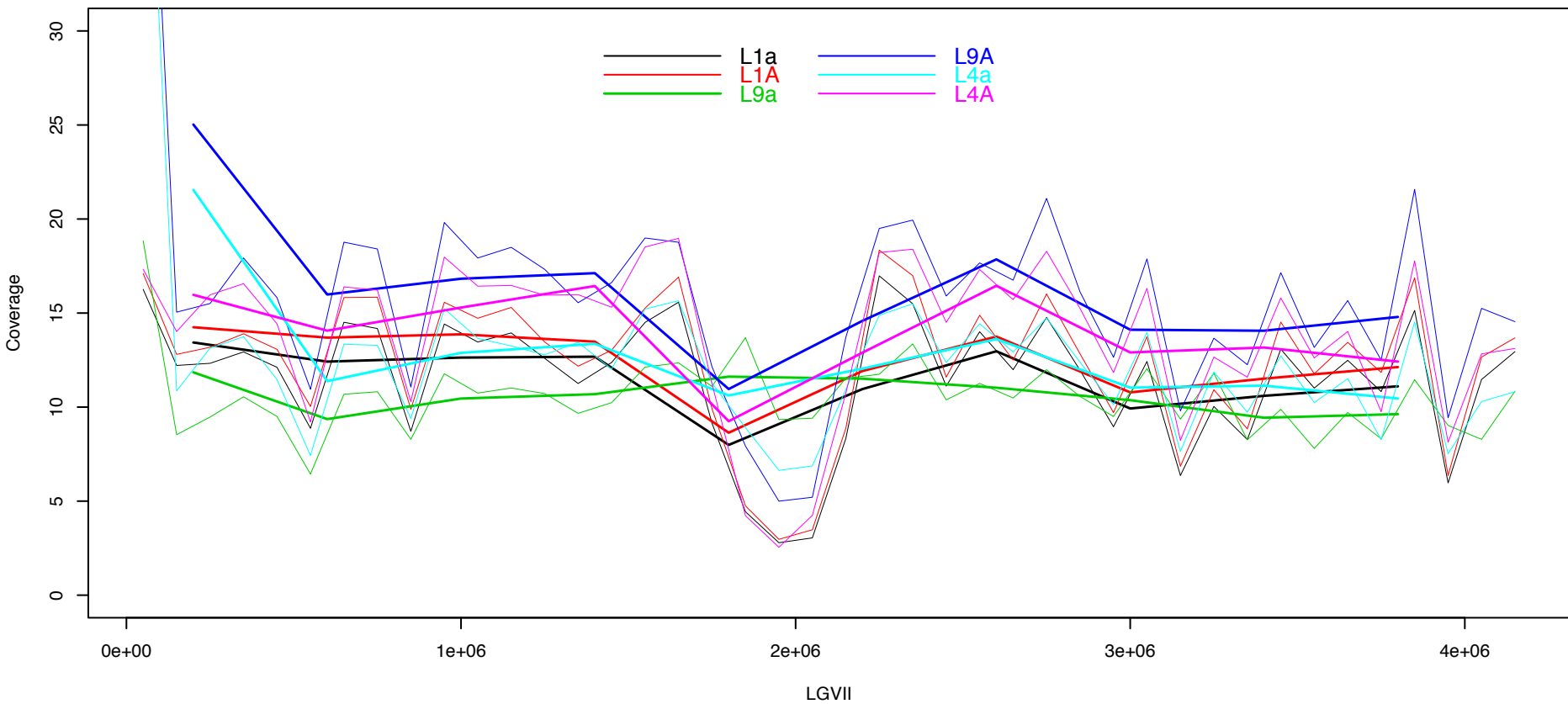

Supplement: Figure S1 — Sliding window coverage depth for seven chromosomes of all six haploid genomes of Neurospora tetrasperma in this study. The thicker lines represent 400 kb window size (step size 400 kb) sliding along the chromosome; thinner lines 100 kb (100 kb). (PDF) [file pgen.1002820.s001.pdf]

Figure S2

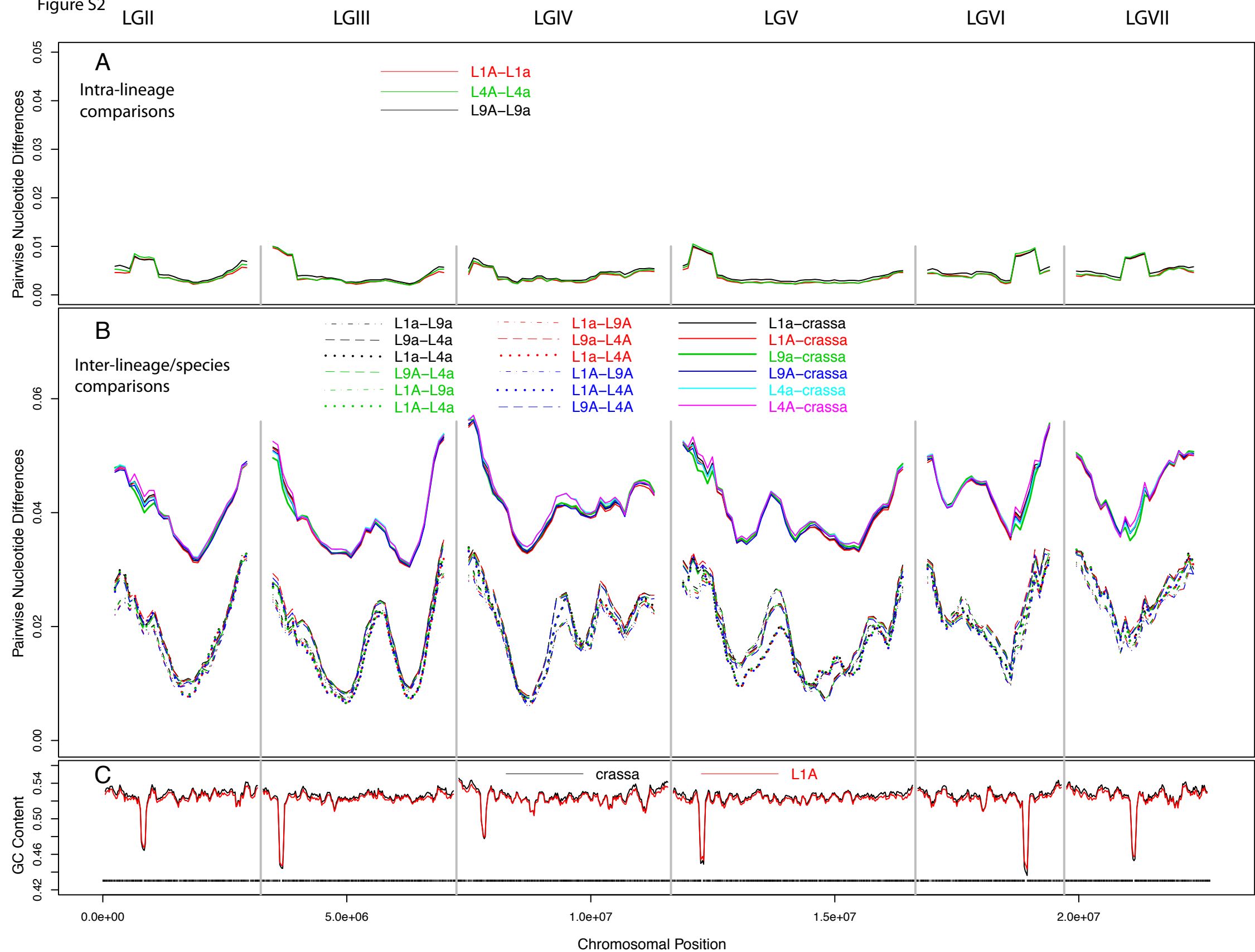

Supplement: Figure S2 — Pair-wise divergences and GC estimates of the six autosomes (LGII-LGVII) of Neurospora tetrasperma and Neurospora crassa. A: Intra-lineage comparisons of N. tetrasperma. The lines represent 500 kb window size (step size 100 kb) sliding along the chromosome, B: Comparisons of genomes from different lineages/species. Solid lines represent N. tetrasperma-N. crassa comparisons and dashed lines represent N. tetrasperma-N. tetrasperma comparisons. All lines represent 500 kb window size (step size 100 kb) sliding along the chromosome. C: GC content in N. crassa (black line) and N. tetrasperma L1A (red line). Window size is 100 kb, step size 20 kb. Black bars indicate gene density in L1A. (PDF) [file pgen.1002820.s002.pdf]

### ***Bml* LGVIL**

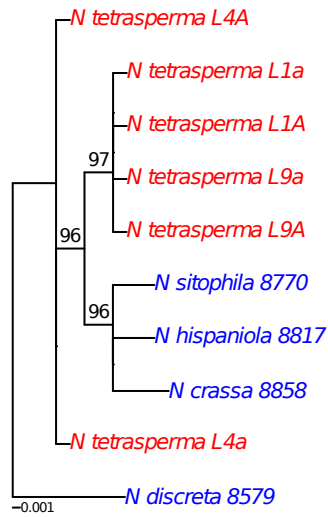

### ***actin* LGVR**

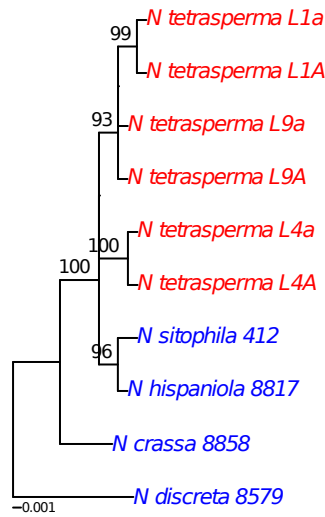

### ***ccg-7* LGIIR**

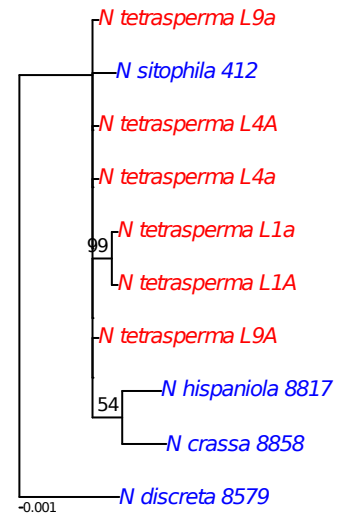

### ***pkc* LGIVR**

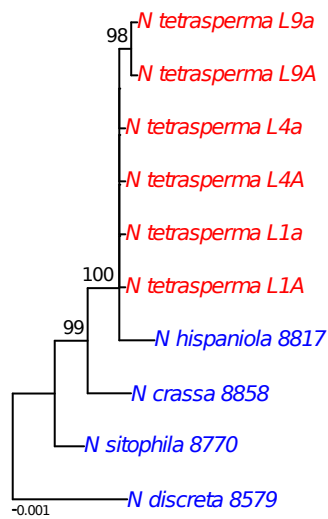

### ***pre-1* LGIIR**

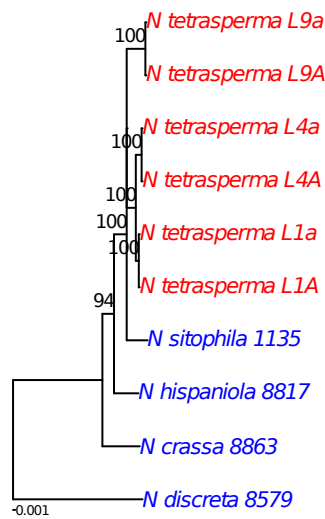

### ***pre-2* LGVIL**

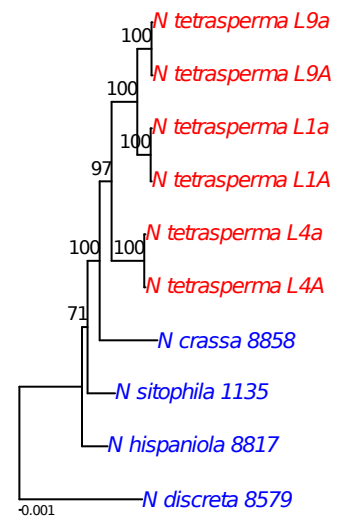

Supplement: Figure S3 — Gene genealogies for six autosomal gene loci of Neurospora. Each tree includes three N. tetrasperma lineages (red), and four heterothallic Neurospora species (blue). Neurospora discreta was used as the outgroup in all analyses. The topologies shown are from the Bayesian phylogenetic reconstruction with the posterior probabilities (as a percentage) from the analysis shown above the branches. (PDF) [file pgen.1002820.s003.pdf]

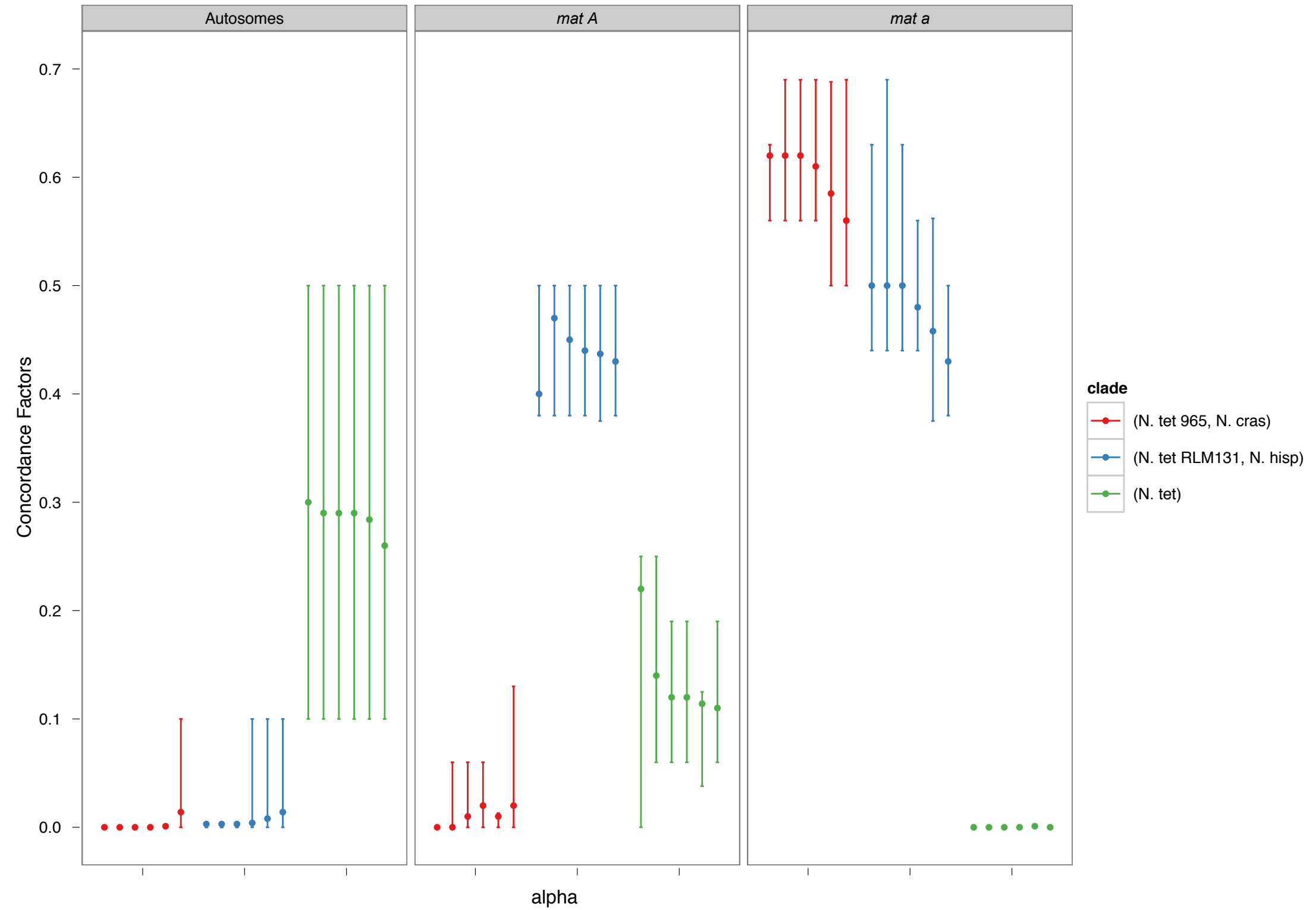

Supplement: Figure S5 — The Concordance Factors of the Bayesian Concordance Analysis for 6 different choices of prior discordance. The alpha priors are from left to right as follows: 0.001, 0.1, 1.0, 10, 100 and ∞. (PDF) [file pgen.1002820.s005.pdf]

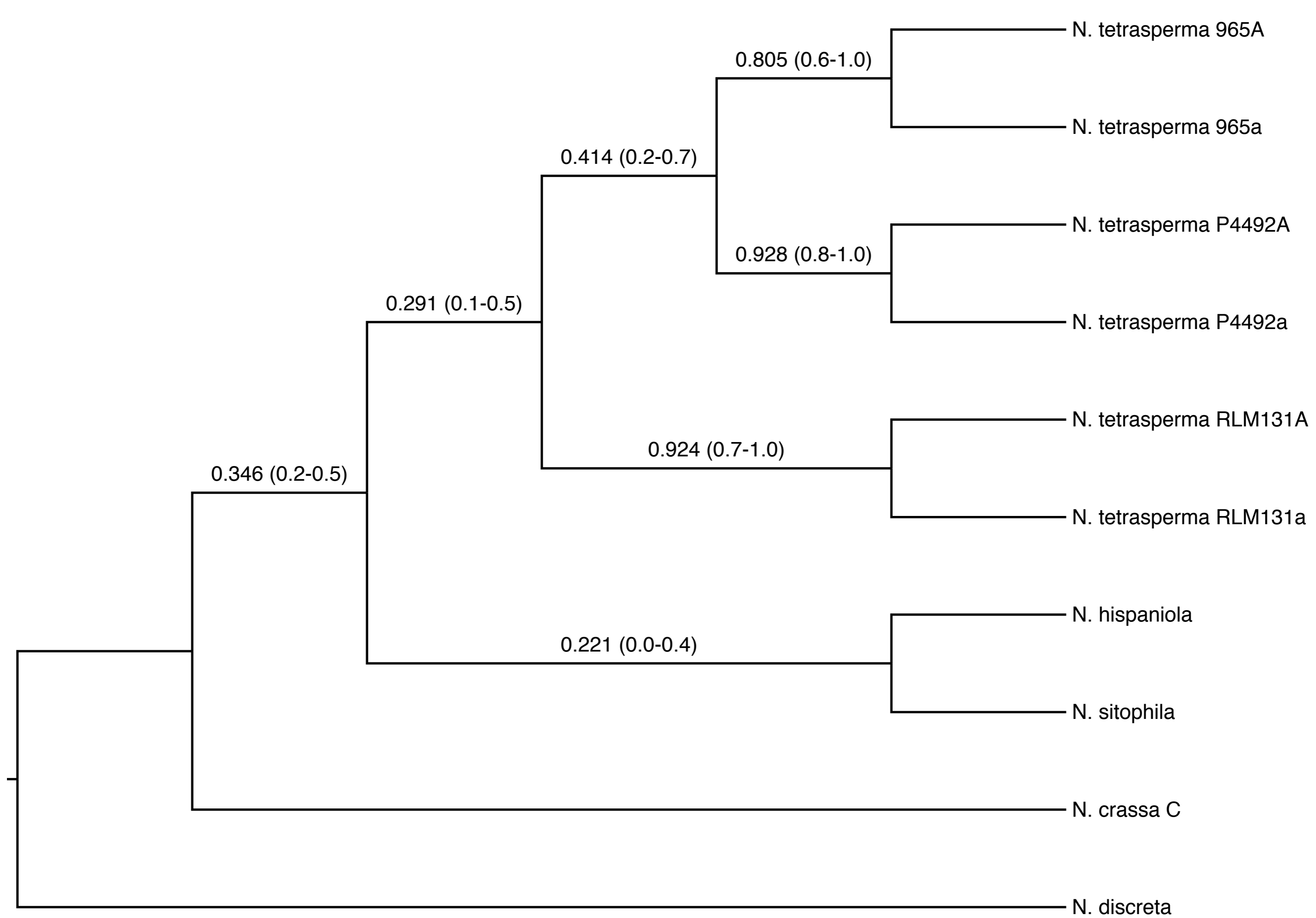

Supplement: Figure S6 — Primary concordance tree for autosomal genes of Neurospora strains included in the BCA. The numbers displayed above the branches represent the concordance factors with the 95% credibility interval in parenthesis. (PDF) [file pgen.1002820.s006.pdf]
