# Supplementary material for: Large-Scale Introgression Shapes the Evolution of the Mating-Type Chromosomes of the Filamentous Ascomycete Neurospora tetrasperma
Source: PLoS Genet. 2012 Jul 26;8(7):e1002820. doi: 10.1371/journal.pgen.1002820 (PMC3406010; doi:10.1371/journal.pgen.1002820)

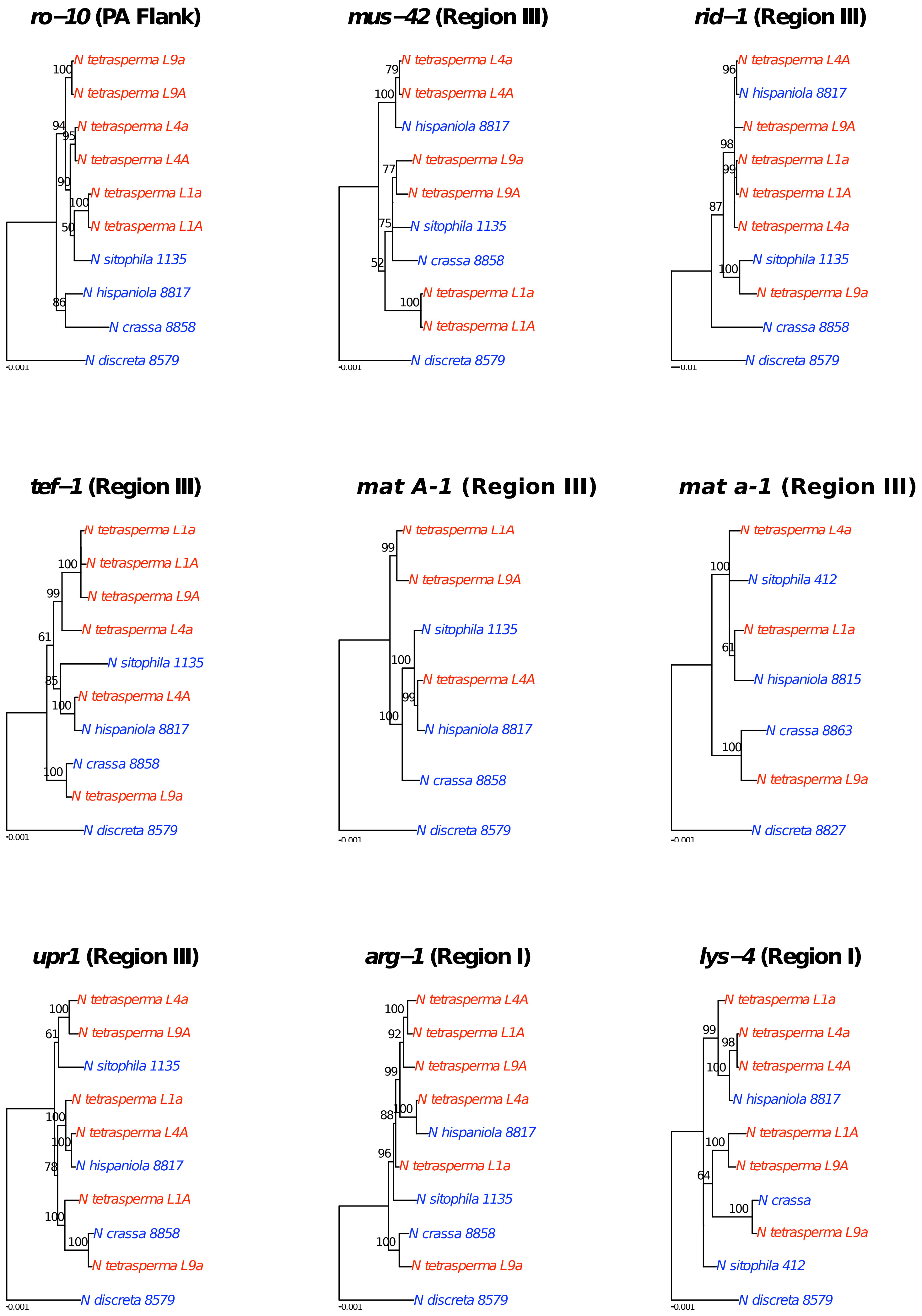
ro–10 (PA Flank)
N tetrasperma L9a
N tetrasperma L9A
N tetrasperma L4a
N tetrasperma L4A
N tetrasperma L1a
N tetrasperma L1A
N sitophila 1135
N hispaniola 8817
N crassa 8858
N discreta 8579
100
94
95
90
100
50
86
0.001
mus–42 (Region III)
N tetrasperma L4a
N tetrasperma L4A
N hispaniola 8817
N tetrasperma L9a
N tetrasperma L9A
N sitophila 1135
N crassa 8858
N tetrasperma L1a
N tetrasperma L1A
N discreta 8579
79
100
77
75
52
100
0.001
rid–1 (Region III)
N tetrasperma L4A
N hispaniola 8817
N tetrasperma L9A
N tetrasperma L1a
N tetrasperma L1A
N tetrasperma L4a
N sitophila 1135
N tetrasperma L9a
N crassa 8858
N discreta 8579
96
98
99
87
100
0.01
tef–1 (Region III)
N tetrasperma L1a
N tetrasperma L1A
N tetrasperma L9A
N tetrasperma L4a
N sitophila 1135
N tetrasperma L4A
N hispaniola 8817
N crassa 8858
N tetrasperma L9a
N discreta 8579
100
99
61
85
100
100
0.001
mat A-1 (Region III)
N tetrasperma L1A
N tetrasperma L9A
N sitophila 1135
N tetrasperma L4A
N hispaniola 8817
N crassa 8858
N discreta 8579
99
100
99
100
0.001
mat a-1 (Region III)
N tetrasperma L4a
N sitophila 412
N tetrasperma L1a
N hispaniola 8815
N crassa 8863
N tetrasperma L9a
N discreta 8827
100
61
100
0.001
upr1 (Region III)
N tetrasperma L4a
N tetrasperma L9A
N sitophila 1135
N tetrasperma L1a
N tetrasperma L4A
N hispaniola 8817
N tetrasperma L1A
N crassa 8858
N tetrasperma L9a
N discreta 8579
100
61
100
100
78
100
100
0.001
arg–1 (Region I)
N tetrasperma L4A
N tetrasperma L1A
N tetrasperma L9A
N tetrasperma L4a
N hispaniola 8817
N tetrasperma L1a
N sitophila 1135
N crassa 8858
N tetrasperma L9a
N discreta 8579
100
92
99
100
88
96
100
0.001
lys–4 (Region I)
N tetrasperma L1a
N tetrasperma L4a
N tetrasperma L4A
N hispaniola 8817
N tetrasperma L1A
N tetrasperma L9A
N crassa
N tetrasperma L9a
N sitophila 412
N discreta 8579
99
98
100
100
64
100
0.001

***ad–9* (Region I)**

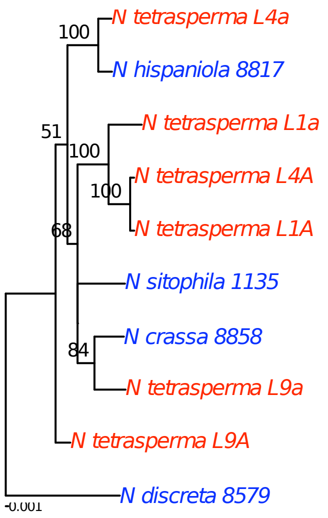

***al–1* (Region II)**

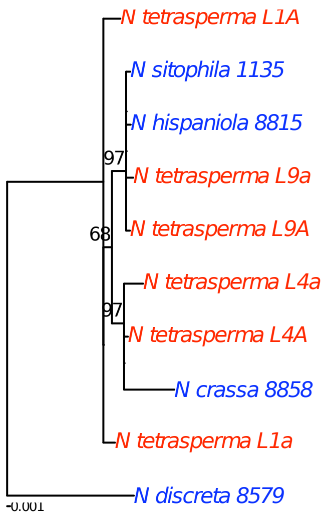

***lys–3* (Region II)**

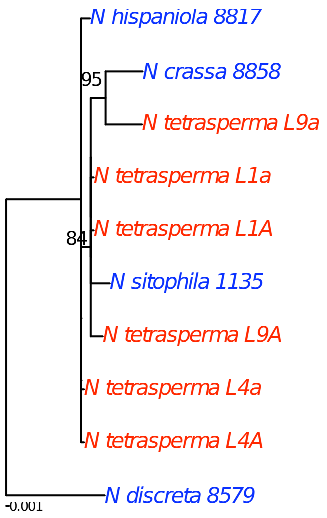

***prd–4* (PA Flank)**

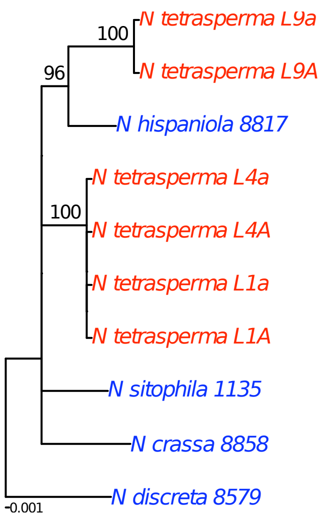

Supplement: Figure S4 — Gene genealogies for 13 genes on the mating-type (mat) chromosome of Neurospora. Each tree includes three N. tetrasperma lineages (red), and four heterothallic Neurospora species (blue). Strain IDs are shown in Table S6. Neurospora discreta was used as the outgroup in all analyses. The topologies shown are from the Bayesian phylogenetic reconstruction with the posterior probabilities (as a percentage) from analysis shown above the branches. (PDF) [file pgen.1002820.s004.pdf]
