# Supplementary material for: Large-Scale Introgression Shapes the Evolution of the Mating-Type Chromosomes of the Filamentous Ascomycete Neurospora tetrasperma
Source: PLoS Genet. 2012 Jul 26;8(7):e1002820. doi: 10.1371/journal.pgen.1002820 (PMC3406010; doi:10.1371/journal.pgen.1002820)
Supplement: Table S1 — Strains of Neurospora used in the study. (PDF) [file pgen.1002820.s007.pdf]

Table S1. Strains of *Neurospora* used in the study.

Species	Strain ID[1]		Mating type	Geographic origin
	Heterokaryon	Homokaryon		
Pseudohomothallic				
N. tetrasperma (L1[3])	P4492	9033[2]	A	Franklin, Louisiana
		9034[2]	a	
N. tetrasperma (L4[3])	RLM131	7585[2]	A	Coba, Mexico
		7586[2]	a	
N. tetrasperma (L9[3])	965	965A[2]	A	Liberia
		965a[2]	a	
Heterothallic				
N. crassa (NcA[4])	n.a.	8900[5,6]	A	Adiopodoume, Ivory Coast
N. crassa (NcA[4])	n.a.	8876[5,6]	a	Franklin, Louisiana
N. crassa (NcB[4])	n.a.	8830[5,6]	A	Golikro, Ivory Coast
N. crassa (NcB[4])	n.a.	8772[5,6]	a	Karnataka, India
N. crassa (NcC[4])	n.a.	8858[5,6,7]	A	Tamil Nadu, India
N. crassa (NcC[4])	n.a.	8863[5,6,7]	a	Tamil Nadu, India
N. discreta	n.a.	8579[5,6,7]	A	Belen, NM
N. discreta	n.a.	8777[6]	A	Texas, USA
N. discreta	n.a.	8827[7]	a	Fogbesso, Ivory Coast
N. hispaniola	n.a.	8817[5,6,7]	A	Carrefour Dufort, Haiti
N. hispaniola	n.a.	8815[5,6,7]	a	Carrefour Dufort, Haiti
N. intermedia (NiA[4])	n.a.	8901[5,6]	A	Monrovia, Liberia
N. intermedia (NiA[4])	n.a.	8869[5,6]	a	Tamil Nadu, India
N. intermedia (NiB[4])	n.a.	8764[5,6]	A	Manila, Phillipines
N. intermedia (NiB[4])	n.a.	8768[5,6]	a	Bogor, Java
N. metzenbergii	n.a.	8880[5,6]	A	Nosy Be, Madagascar
N. metzenbergii	n.a.	8853[5,6]	a	Yukatan, Mexico
N. perkinsii	n.a.	8838[5,6]	A	Madingo, Congo
N. perkinsii	n.a.	8835[5,6]	a	Makaba, Congo
N. sitophila	n.a.	1135[5,7]	A	Panama
N. sitophila	n.a.	10394[6]	a	Laie, Hawaii

N. sitophila	n.a.	412[5,7]	a	Unknown
N. sitophila	n.a.	8770[7]	A	Karnataka, India
N. sitophila	n.a.	10409[7]	A	Chemax, Mexico

[1]IDs are Fungal Genetic Stock Center (FGSC), Perkins (P) or RL Metzenberg (RLM) numbers. For *N. tetrasperma*: the two homokaryotic, single mating-type component strains are derived from the listed heterokaryon. n.a.; non applicable (heterothallic *Neurospora* grows in nature as homokaryotic/haploid).

[2]Strains used for Illumina sequencing

[3]Phylogenetic lineages described in Menkis et al. (2009), *Journal of Evolutionary Biology* 22(9): 1923-1936

[4]Phylogenetic subgroup designated by Dettman et al. (2003), *Evolution* 57(12): 2703-2720

[5]Strains used in phylogenetic analyses of genes along the *mat* chromosome

[6]Strains used in phylogenetic analyses of autosomal microsatellite flanking loci

[7]Strains used for Bayesian Concordance Analyses (BCA)
