# Supplementary material for: Large-Scale Introgression Shapes the Evolution of the Mating-Type Chromosomes of the Filamentous Ascomycete Neurospora tetrasperma
Source: PLoS Genet. 2012 Jul 26;8(7):e1002820. doi: 10.1371/journal.pgen.1002820 (PMC3406010; doi:10.1371/journal.pgen.1002820)
Supplement: Table S2 — Assembly statistics for six haploid genomes of Neurospora tetrasperma. (PDF) [file pgen.1002820.s008.pdf]

Table S2. Assembly statistics for six haploid genomes of *Neurospora tetrasperma*

Strain	Total paired-end reads	Total paired-end reads aligned	Total basepair aligned	Average Error Probability[a]	Coverage ratio (%) of reference genome	Average depth for *mat* chromosome	Average depth for whole genome
L1A	7,631,959	5,348,236	518,083,893	<0.0020	80.6	13.8X	15.1X
L1a	6,127,564	4,940,974	479,947,319	<0.0012	80.1	12.8X	13.9X
L4A	6,924,278	5,938,125	573,472,938	<0.0012	79.6	15.6X	16.2X
L4a	6,782,849	4,971,470	469,579,413	<0.0020	81.6	13.9X	14.9X
L9A	7,682,941	6,529,667	631,361,064	<0.0020	81.7	17.7X	19.2X
L9a	5,019,911	4,081,623	386,796,068	<0.0030	84.1	11.7X	12.0X

[a]The average error rate for each nucleotide in the sequenced genome, calculated based on Average reads quality using the formula Qsolexa = $-10 \times \log_{10}(Pe/1-Pe)$.
