# Supplementary material for: Large-Scale Introgression Shapes the Evolution of the Mating-Type Chromosomes of the Filamentous Ascomycete Neurospora tetrasperma
Source: PLoS Genet. 2012 Jul 26;8(7):e1002820. doi: 10.1371/journal.pgen.1002820 (PMC3406010; doi:10.1371/journal.pgen.1002820)
Supplement: Table S3 — Pair-wise nucleotide differences, estimated as the fraction of different nucleotides, for the mat chromosome and autosomes of six haploid genomes of Neurospora tetrasperma, and Neurospora crassa. (PDF) [file pgen.1002820.s009.pdf]

Table S3. Pair-wise nucleotide differences, estimated as the fraction of different nucleotides, for the *mat* chromosome and autosomes of six haploid genomes of *Neurospora tetrasperma*, and *N. crassa*.

Pair-wise comparison	Chromosome						
	mat chromosome	Autosomes					
	LGI	LGII	LGIII	LGIV	LGV	LGVI	LGVII
Within *N. tetrasperma* lineages							
L1a-L1A	0.0148	0.0044	0.0039	0.0038	0.0037	0.0048	0.0049
L9a-L9A	0.0277	0.0052	0.0044	0.0044	0.0042	0.0053	0.0055
L4a-L4A	0.0195	0.0047	0.0041	0.004	0.0038	0.0049	0.0051
Between *N. tetrasperma* lineages							
L1a-L9a	0.0337	0.0194	0.0175	0.0196	0.0197	0.0232	0.0246
L1a-L9A	0.0238	0.0188	0.0171	0.0191	0.0192	0.0225	0.0241
L1A-L9a	0.0337	0.0193	0.0173	0.0195	0.0196	0.023	0.0244
L1A-L9A	0.0200	0.0187	0.0168	0.0189	0.0191	0.0223	0.0239
L1a-L4a	0.0256	0.0206	0.0172	0.0211	0.0186	0.0234	0.0268
L1a-L4A	0.0242	0.0204	0.017	0.0208	0.0184	0.0232	0.0266
L1A-L4a	0.0262	0.0204	0.017	0.021	0.0184	0.0231	0.0267
L1A-L4A	0.0205	0.0202	0.0168	0.0206	0.0182	0.023	0.0264
L9a-L4a	0.0353	0.0209	0.0195	0.0222	0.0202	0.0244	0.0272
L9a-L4A	0.0351	0.0209	0.0195	0.0221	0.0202	0.0245	0.0272
L9A-L4a	0.0271	0.0204	0.0192	0.0217	0.0198	0.024	0.0269
L9A-L4A	0.0223	0.0203	0.0191	0.0215	0.0196	0.0239	0.0267
N. crassa – N. tetrasperma							
N.crassa-L1a	0.0381	0.0411	0.0395	0.0424	0.041	0.0455	0.0452
N.crassa-L1A	0.0380	0.0409	0.0393	0.0421	0.0407	0.0451	0.0449
N.crassa-L9a	0.0319	0.0411	0.0394	0.0426	0.0411	0.0454	0.0452

N.crassa-L9A	0.0380	0.041	0.0394	0.0423	0.0409	0.0454	0.0452
N.crassa-L4a	0.0388	0.0413	0.0398	0.043	0.0415	0.0456	0.0452
N.crassa-L4A	0.0387	0.0415	0.04	0.0431	0.0415	0.0458	0.0454
