# Supplementary material for: Large-Scale Introgression Shapes the Evolution of the Mating-Type Chromosomes of the Filamentous Ascomycete Neurospora tetrasperma
Source: PLoS Genet. 2012 Jul 26;8(7):e1002820. doi: 10.1371/journal.pgen.1002820 (PMC3406010; doi:10.1371/journal.pgen.1002820)
Supplement: Table S4 — Boundaries and nucleotide divergences for regions of the mating-type (mat) chromosomes originating from three Neurospora tetrasperma wild-type heterokaryons. (PDF) [file pgen.1002820.s010.pdf]

Table S4. Boundaries and nucleotide divergences for regions of the mating-type (*mat*) chromosomes originating from three *Neurospora tetrasperma* wild-type heterokaryons.

Strain ID	Pseudoautosomal (PA) region 1		Region of elevated sequence divergence		Pseudoautosomal (PA) region 2		Introgression tract
	Position	Nucleotide divergence	Position	Nucleotide divergence	Position	Nucleotide divergence	Position
L1	1-1,390,000	0.0065	1,390,001-6,760,000	0.0187	6,760,001-7,657,888	0.0044	1,400,001-5,490,000
L4	1-1,140,000	0.0062	1,140,001-7,000,000	0.0238	7,000,001-7,657,888	0.0043	1,500,001-6,710,000
L9	1-900,000	0.0068	900,001-6,780,000	0.0343	6,780,001-7,657,888	0.0054	1,120,001-6,710,000
