# Supplementary material for: Large-Scale Introgression Shapes the Evolution of the Mating-Type Chromosomes of the Filamentous Ascomycete Neurospora tetrasperma
Source: PLoS Genet. 2012 Jul 26;8(7):e1002820. doi: 10.1371/journal.pgen.1002820 (PMC3406010; doi:10.1371/journal.pgen.1002820)
Supplement: Table S5 — Pair-wise nucleotide differences, estimated as the fraction of different nucleotides, of different regions of the mating-type (mat) chromosome (LGI). Data from pseudoautosomal (PA) regions are given as the PA regions shared between all heterokaryons of N. tetrasperma PA1: (position 1–900,000), the central region introgressed in all three heterokaryons (1,480,000–5,450,000) and PA2 shared between the heterokaryons (7,000,000–7,657,888). (PDF) [file pgen.1002820.s011.pdf]

Table S5. Pair-wise nucleotide differences, estimated as the fraction of different nucleotides, of different regions of the mating-type (*mat*) chromosome (LGI). Data from pseudoautosomal (PA) regions are given as the PA regions shared between all heterokaryons of *N. tetrasperma* PA1: (position 1-900,000), the central region introgressed in all three heterokaryons (1,480,000-5,450,000) and PA2 shared between the heterokaryons (7,000,000-7,657,888).

Pair-wise comparison	*mat* chromosome (LGI)		
	PA1	central	PA2
Between *N. tetrasperma* lineages			
L1a-L9a	0.0266	0.0360	0.0265
L1a-L9A	0.0258	0.0240	0.0258
L1A-L9a	0.0262	0.0362	0.0263
L1A-L9A	0.0254	0.0169	0.0256
L1a-L4a	0.0292	0.0240	0.0272
L1a-L4A	0.0289	0.0237	0.0269
L1A-L4a	0.0289	0.0255	0.0271
L1A-L4A	0.0286	0.0166	0.0268
L9a-L4a	0.0315	0.0370	0.0292
L9a-L4A	0.0311	0.0370	0.0289
L9A-L4a	0.0308	0.0264	0.0285
L9A-L4A	0.0305	0.0183	0.0282
N. crassa – N. tetrasperma			
N.crassa-L1a	0.0440	0.0354	0.0443
N.crassa-L1A	0.0438	0.0355	0.0440
N.crassa-L9a	0.0443	0.0273	0.0446
N.crassa-L9A	0.0439	0.0357	0.0442
N.crassa-L4a	0.0447	0.0363	0.0447
N.crassa-L4A	0.0446	0.0363	0.0447
