# Supplementary material for: Large-Scale Introgression Shapes the Evolution of the Mating-Type Chromosomes of the Filamentous Ascomycete Neurospora tetrasperma
Source: PLoS Genet. 2012 Jul 26;8(7):e1002820. doi: 10.1371/journal.pgen.1002820 (PMC3406010; doi:10.1371/journal.pgen.1002820)
Supplement: Table S6 — Information on loci and heterothallic strains of Neurospora used in gene tree and Bayesian Concordance analyses. (PDF) [file pgen.1002820.s012.pdf]

Table S6. Information on loci and heterothallic strains of *Neurospora* used in gene tree and Bayesian Concordance analyses.

Locus[1]			Strain ID[2]			
	Genomic Location	Length (bp)	*N. crassa*	*N. sitophila*	*N. hispaniola*	*N. discreta*
Microsatellite flanking loci	Autosome					
DMG[3,4]	LGIVR	333	8858	10409	8817	8579
QMA[3,4]	LGIIR	337	8858	10409	8817	8579
TMI[3,4]	LGVR	416	8858	10409	8817	8579
TML[3,4]	LGVL	474	8858	10409	8817	8579
Nuclear gene loci						
Bml [5]	LGVIL	513	8858	8770	8817	8579
actin[5]	LGVR	808	8858	412	8817	8579
ccg7 [5]	LGIIR	753	8858	412	8817	8579
pkc[5]	LGIVR	557	8858	8770	8817	8579
pre-1[5]	LGIIIR	2248	8863	1135	8817	8579
pre-2[5]	LGVIIL	1350	8858	1135	8817	8579
	mat-chromosome[7]					
ro-10 [6]		619	8858	1135	8817	8579
nit-2[6]		606	8858	1135	8817	8579
mus-42[6]		775	8858	1135	8817	8579
rid-1 [6]		733	8858	1135	8817	8579
tef-1[6]		752	8858	1135	8817	8579
mat A-1 [6]		990	8858	1135	8817	8579
mat a-1 [6]		793	8863	412	8815	8827
upr-1 [6]		1018	8858	1135	8817	8579
arg-1[6]		1589	8858	1135	8817	8579
eth-1[6]		918	8858	1135	8817	8579
lys-4[6]		680	8858	412	8817	8579
cys-9[6]		743	8858	1135	8815	8579
ad-9[6]		595	8858	1135	8817	8579
al-1[6]		837	8858	1135	8815	8579
lys-3[6]		961	8858	1135	8817	8579
prd-4 [6]		752	8858	1135	8817	8579
phr [6]		1786	8858	412	8815	8579

[1]Locus name for microsatellite loci as in Dettman, J. R., and J. W. Taylor. (2004). *Genetics* 168:1231-1248, and locus name for genes as in the *Neurospora* compendium on chromosomal loci (http://www.fgsc.net/2000compendium/2000compend.html). [2]Fungal Genetics Stock Center (FGSC) number, detailed information of strains is given in Table S1. Sequence data from [3]Dettman et al. (2003), *Evolution* 57(12): 2703-2720; [4]Villalta et al. (2009), Mycologia, 101(6), 2009, pp. 777–789; [5]Strandberg et al. (2010) *Fungal Genetics and Biology* 47: 869–878; [6]generated specifically for this study, with the exception of N. discreta

for which the data originate from the Joint Genome Institute (http://www.jgi.doe.gov/); [7]For specific location, see Figure 3.
