# Supplementary material for: Large-Scale Introgression Shapes the Evolution of the Mating-Type Chromosomes of the Filamentous Ascomycete Neurospora tetrasperma
Source: PLoS Genet. 2012 Jul 26;8(7):e1002820. doi: 10.1371/journal.pgen.1002820 (PMC3406010; doi:10.1371/journal.pgen.1002820)
Supplement: Table S7 — Concordance factor (CF) and 95% credibility interval estimated for clades in BCA analysis of autosomal loci. The table shows only clades with concordance factor greater than 0.05. (PDF) [file pgen.1002820.s013.pdf]

Table S7. Concordance factor (CF) and 95% credibility interval estimated for clades in BCA analysis of autosomal loci. The table shows only clades with concordance factor greater than 0.05.

Autosomes		
Clade*	CF	95% Credibility
{1,2,3,4,5,6,9,10|7,8}	0.928	(0.800,1.000)
{1,2,3,4,5,6,7,8|9,10}	0.924	(0.700,1.000)
{1,2|3,4,5,6,7,8,9,10}	0.805	(0.600,1.000)
{1,2,7,8|3,4,5,6,9,10}	0.414	(0.200,0.700)
{1,2,5,6,7,8,9,10|3,4}	0.346	(0.200,0.500)
{1,2,7,8,9,10|3,4,5,6}	0.291	(0.100,0.500)
{1,2,3,4,7,8,9,10|5,6}	0.221	(0.000,0.400)
{1,2,4,6,7,8,9,10|3,5}	0.268	(0.100,0.500)
{1,2,3,5,7,8,9,10|4,6}	0.246	(0.200,0.500)
{1,2,5,7,8|3,4,6,9,10}	0.204	(0.200,0.300)
{1,2,5,7,8,9,10|3,4,6}	0.202	(0.200,0.300)
{1,2,6,7,8,9,10|3,4,5}	0.202	(0.100,0.400)
{1,2,5|3,4,6,7,8,9,10}	0.189	(0.000,0.200)
{1,2,3,4,5,6|7,8,9,10}	0.185	(0.100,0.300)
{1,2,4,6,7,8|3,5,9,10}	0.184	(0.100,0.300)
{1,2,3,5,6,7,8|4,9,10}	0.136	(0.000,0.300)
{1,2,4,7,8,9,10|3,5,6}	0.136	(0.000,0.300)
{1,2,3,7,8,9,10|4,5,6}	0.120	(0.100,0.200)
{1,2,5,6,7,8|3,4,9,10}	0.092	(0.000,0.200)
{1,2,3,5,6,9,10|4,7,8}	0.07	(0.000,0.300)
{1,2,3,6,7,8,9,10|4,5}	0.064	(0.000,0.200)
{1,7,8|2,3,4,5,6,9,10}	0.054	(0.000,0.300)
{1,3,4,5,6,9,10|2,7,8}	0.051	(0.000,0.300)

* Taxon Number: 1. N. tetrasperma 965A, 2. N. tetrasperma 965a, 3. N. crassa, 4. N. discreta, 5. N. hispaniola, 6. N. sitophila, 7. N. tetrasperma P4492A, 8. N. tetrasperma P4492a, 9. N. tetrasperma RLM131A, 10. N. tetrasperma RLM131a.
