# Supplementary material for: Large-Scale Introgression Shapes the Evolution of the Mating-Type Chromosomes of the Filamentous Ascomycete Neurospora tetrasperma
Source: PLoS Genet. 2012 Jul 26;8(7):e1002820. doi: 10.1371/journal.pgen.1002820 (PMC3406010; doi:10.1371/journal.pgen.1002820)
Supplement: Table S8 — Concordance factor (CF) and 95% credibility interval estimated for clades in BCA analysis of loci on the mating-type (mat) chromosomes. The table shows only clades with concordance factors greater than 0.05. (PDF) [file pgen.1002820.s014.pdf]

Table S8. Concordance factor (CF) and 95% credibility interval estimated for clades in BCA analysis of loci on the mating-type (*mat*) chromosomes. The table shows only clades with concordance factors greater than 0.05.

mat A chromosome			*mat a* chromosome		
Clade*	CF	95% Credibility interval	Clade*	CF	95% Credibility interval
{1,2,3,5,6|4,7}	0.453	(0.375,0.500)	{1,2|3,4,5,6,7}	0.616	(0.562,0.688)
{1,4,5,6,7|2,3}	0.450	(0.250,0.625)	{1,2,3|4,5,6,7}	0.581	(0.375,0.625)
{1,6|2,3,4,5,7}	0.305	(0.188,0.438)	{1,2,3,5,6|4,7}	0.496	(0.438,0.625)
{1,4,6,7|2,3,5}	0.231	(0.062,0.375)	{1,2,3,5|4,6,7}	0.476	(0.062,0.625)
{1,2,3,4,5|6,7}	0.264	(0.188,0.312)	{1,2,3,5,7|4,6}	0.169	(0.062,0.250)
{1,2,3,4|5,6,7}	0.239	(0.188,0.250)	{1,2,3,4,6|5,7}	0.147	(0.062,0.250)
{1,4|2,3,5,6,7}	0.205	(0.125,0.312)	{1,2,3,4,5|6,7}	0.129	(0.062,0.250)
{1,2,3,6|4,5,7}	0.182	(0.125,0.250)	{1,2,4,5,7|3,6}	0.117	(0.062,0.188)
{1,3,6|2,4,5,7}	0.180	(0.125,0.250)	{1,5|2,3,4,6,7}	0.116	(0.062,0.250)
{1,6,7|2,3,4,5}	0.118	(0.062,0.188)	{1,3,4,5,6|2,7}	0.113	(0.062,0.188)
{1,5|2,3,4,6,7}	0.115	(0.000,0.250)	{1,4,5,6,7|2,3}	0.108	(0.000,0.250)
{1,7|2,3,4,5,6}	0.105	(0.062,0.188)	{1,2,3,6,7|4,5}	0.097	(0.000,0.188)
{1,3,4,5,6|2,7}	0.096	(0.000,0.188)	{1,2,3,4|5,6,7}	0.087	(0.000,0.188)
{1,2,3,6,7|4,5}	0.084	(0.000,0.188)	{1,4|2,3,5,6,7}	0.069	(0.000,0.125)
{1,3,4,5,7|2,6}	0.080	(0.062,0.125)	{1,5,6,7|2,3,4}	0.066	(0.000,0.125)
{1,2,4,5,7|3,6}	0.077	(0.000,0.188)	{1,3,5,6,7|2,4}	0.063	(0.000,0.125)
{1,4,5,6|2,3,7}	0.075	(0.000,0.125)	{1,4,5|2,3,6,7}	0.063	(0.000,0.125)
{1,3,5,6,7|2,4}	0.075	(0.000,0.188)	{1,2,3,6|4,5,7}	0.051	(0.000,0.375)
{1,5,6,7|2,3,4}	0.068	(0.000,0.125)			
{1,4,5|2,3,6,7}	0.060	(0.000,0.062)			
{1,2,3,5|4,6,7}	0.058	(0.000,0.250)			
{1,2,5,6|3,4,7}	0.057	(0.000,0.125)			

*Taxon Number : 1. N. tetrasperma L9, 2. N. crassa, 3. N. discreta, 4. N. hispaniola, 5. N. sitophila, 6. N. tetrasperma L1, 7. N. tetrasperma L4.
