# Supplementary material for: Large-Scale Introgression Shapes the Evolution of the Mating-Type Chromosomes of the Filamentous Ascomycete Neurospora tetrasperma
Source: PLoS Genet. 2012 Jul 26;8(7):e1002820. doi: 10.1371/journal.pgen.1002820 (PMC3406010; doi:10.1371/journal.pgen.1002820)
Supplement: Table S9 — The preferred codons for Neurospora tetrasperma and Neurospora crassa. (PDF) [file pgen.1002820.s015.pdf]

Table S9. The preferred codons for *Neurospora tetrasperma* and *N. crassa*.

Amino Acid	Preferred Codons
Ala	GCC
Arg	AGG
Arg	CGC
Asn	AAC
Asp	GAC
Cys	TGC
Gln	CAG
Glu	GAG
Gly	GGC
His	CAC
Ile	ATC
Leu	CTC
Leu	CTG
Lys	AAG
Phe	TTC
Pro	CCC
Ser	TCC
Ser	AGC
Thr	ACC
Tyr	TAC
Val	GTC
